# Supplementary material for: Allosteric regulation in STAT3 interdomains is mediated by a rigid core: SH2 domain regulation by CCD in D170A variant
Source: PLoS Comput Biol. 2022 Dec 21;18(12):e1010794. doi: 10.1371/journal.pcbi.1010794 (PMC9815575; doi:10.1371/journal.pcbi.1010794)
Supplement: S1 Text — D170A mutation induces large structural but minor dynamical changes in STAT3.Table A. Statistical differences for the α3 global tilt among different macrostates (Fig 3F). Kolmogorov-Smirnov test and T-test were done using scipy.stats.ks_2samp and scipy.stats.ttest_ind function respectively. (DOCX) [file pcbi.1010794.s001.docx]

Supplemental Information

**﻿**Allosteric Regulation in STAT3 Interdomains is Mediated by a Rigid Core: SH2 Domain Regulation by CCD in D170A Variant

Tingting Zhao, Nischal Karki, Brian D. Zoltowski, Devin A. Matthews*

Department of Chemistry, Southern Methodist University, Dallas, Texas 75275, USA

[dmatthews@smu.edu](mailto:dmatthews@smu.edu)

# Supplementary Results

## **D170A mutation induces large structural but minor dynamical changes in STAT3**

A Root Mean Square Deviation (RMSD) cross-correlation analysis between different domains shows a distinct increase in correlation between the CCD and SH2 domains in the D170A variant compared to the wild-type (S11A and S11B Fig). This correlation is enhanced at the expense of correlation of different domains to the linker domain, with only CCD retaining its correlations with linker in D170A variant.

Using the crystal structure (PDB ID: 6TLC) as the reference, RMSD values of wild type and the D170A variant were computed and shown in a violin plot (S11A Fig). The distribution of RMSD values demonstrates that the D170A variant has multiple quasi-stable conformations seen as multiple peaks in the core full length protein violin plot, whereas the wild type consists of a single pronounced peak. Investigation of each domain shows similar trends, with SH2 having the largest difference between wild type and mutant due to significantly higher RMSD values in D170A, and with LD showing the least change upon mutation. The RMSD distribution of SH2 domain in the six replicas for D170A variant were also plotted (S11D Fig), the multiple quasi-stable conformations in S11C Fig were observed in a few simulations, with the others explore one stable conformation, which is consistent with the observation form pair RMSD plots that the simulations have either equilibrated into a local minimum or transition between local minimums. Overall, the deviation of the SH2 domain from the crystal structure is significantly increased and several quasi-stable conformations appear, while the CCD domain explore similar landscape with crystal structure. However, the CCD domain also exhibits a minority configuration with increased deviation from the crystal structure. This minority configuration corresponds to a kinked configuration of the α1 helix, as is described in our clustering analysis (Section 2.2).

Root Mean Square Fluctuation (RMSF) analysis shows that the difference in conformational dynamics between the wild type and the D170A variant is statistically insignificant in our sample set, even though the overall structural conformations of the two variants are quite different. Qualitative observation of the mean RMSF values shows only slight differences in each of the domains. Specifically, small increases in flexibility are seen in the α1–α2 and α4–α5 loops in CCD, as well as the β11, β11–β12, β13–β14, and β14–α15 loops in DBD. Inversely, flexibility in the D170A mutant is slightly decreased in the β22, α23–α24 loop, α24–β25 loop, and β25 in LD, and the β30–β31 and α33–34 loops in SH2 domain (S6 Fig). Even though the changes in overall flexibility are small, the D170A variant experiences significant changes in the number and geometry of the available quasi-stable conformations within each domain as well as the whole protein. The source of these conformational changes is explored in the following sections.

# Supplementary Tables

|  | Kolmogorov-Smirnov test | | T-test | | |
| --- | --- | --- | --- | --- | --- |
|  | statistic | P-value | Degree of freedom | statistic | P-value |
| M0 vs M1 | 0.198308 | <0.001 | 7028 | 20.19042 | <0.001 |
| M0 vs M2 | 0.475441 | 0.336959 | 6882 | 57.96138 | <0.001 |
| M0 vs M3 | 0.265266 | <0.001 | 6610 | 26.0582 | <0.001 |
| M0 vs M4 | 0.366955 | <0.001 | 3931 | 49.1721 | <0.001 |
| M1 vs M2 | 0.33437 | 0.005855 | 12019 | 44.35194 | <0.001 |
| M1 vs M3 | 0.074214 | <0.001 | 11219 | 6.581899 | <0.001 |
| M1 vs M4 | 0.189818 | <0.001 | 7990 | 32.10457 | <0.001 |
| M2 vs M3 | 0.298892 | <0.001 | 10969 | -38.6448 | <0.001 |
| M2 vs M4 | 0.161786 | <0.001 | 7725 | -26.4637 | <0.001 |
| M3 vs M4 | 0.143099 | <0.001 | 6799 | 24.44831 | <0.001 |

## **Table A:** Statistical differences for the α3 global tilt among different macrostates (Fig 3F). Kolmogorov-Smirnov test and T-test were done using scipy.stats.ks_2samp and scipy.stats.ttest_ind function respectively.
