# Supplementary figures and images for: Allosteric regulation in STAT3 interdomains is mediated by a rigid core: SH2 domain regulation by CCD in D170A variant
Source: PLoS Comput Biol. 2022 Dec 21;18(12):e1010794. doi: 10.1371/journal.pcbi.1010794 (PMC9815575; doi:10.1371/journal.pcbi.1010794)

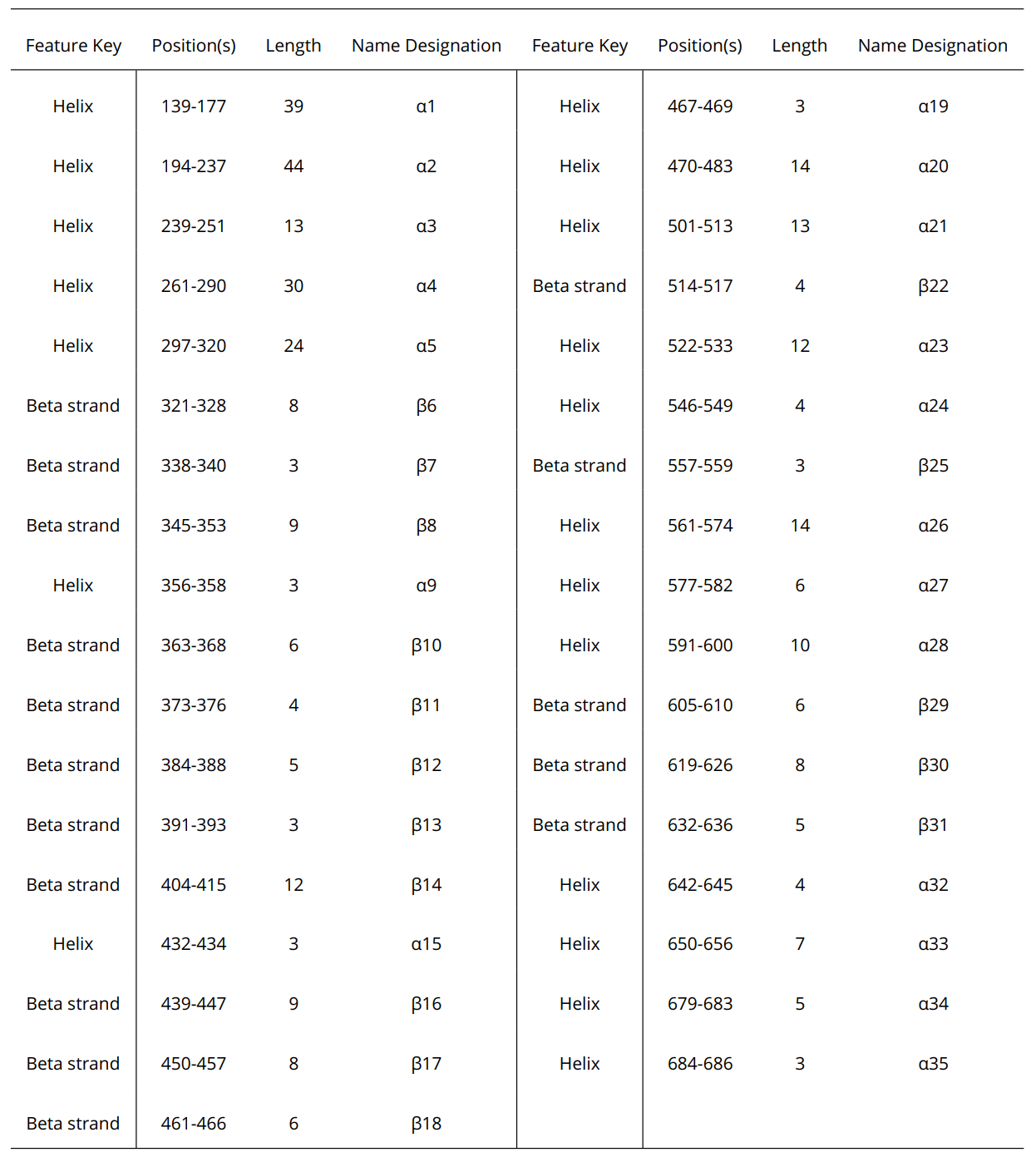

Supplement: S1 Table — (TIF) [file pcbi.1010794.s002.tif]

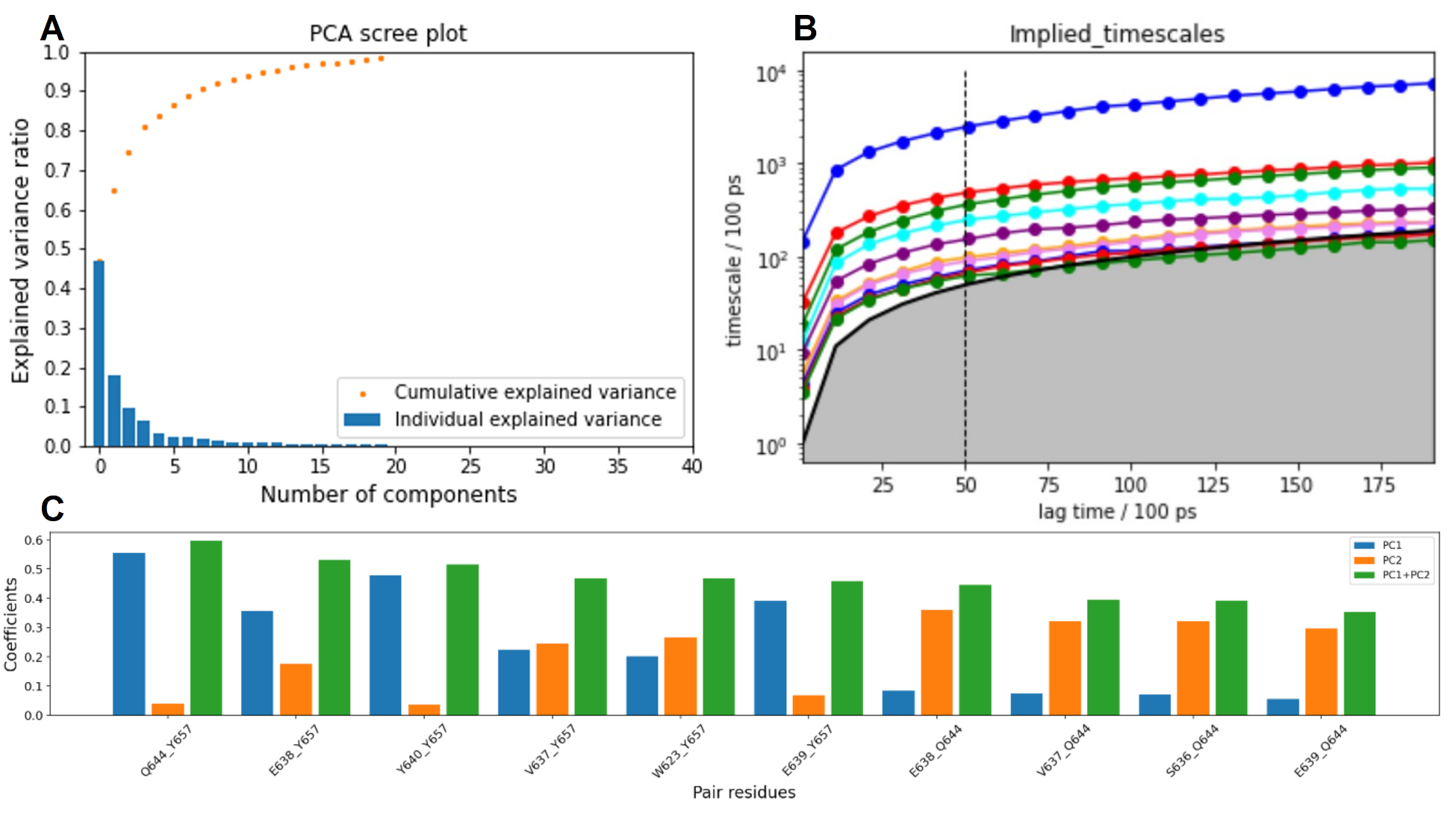

Supplement: S1 Fig — (A) PCA scree plot: dot shows the cumulative explained variance of the principal components; the bar chart represents the explained values per component. (B) Relaxation timescales of MSM for SH2 domain conformational space at different lag times. (C) The first ten features that contribute the first two PC the most. The absolute value of PC1 and PC2, and square root of sum of squared PC1 and PC2 values are shown here. (TIF) [file pcbi.1010794.s003.tif]

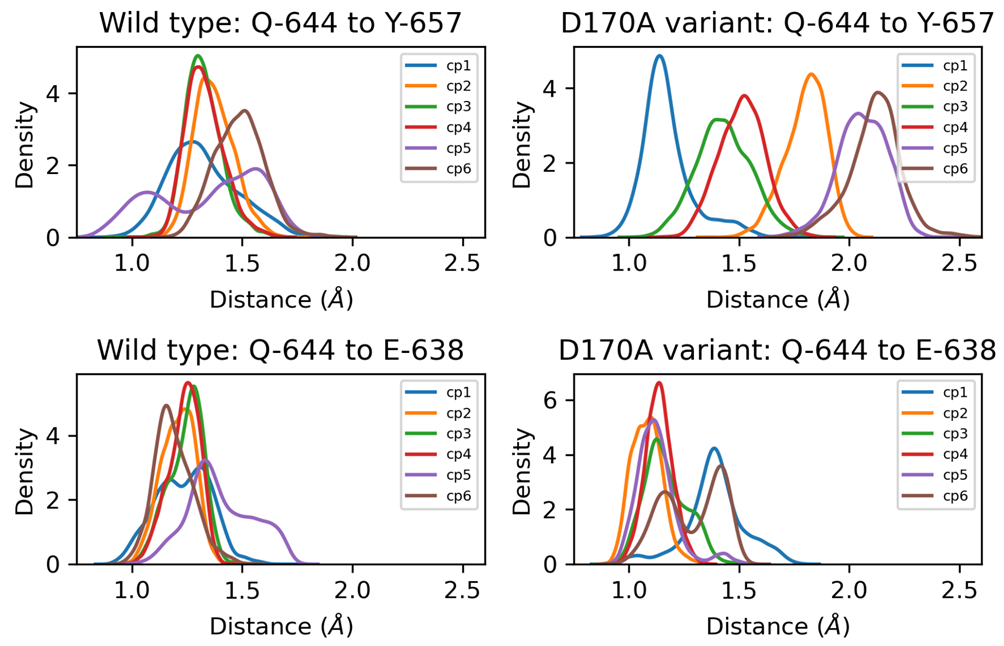

Supplement: S2 Fig — (TIF) [file pcbi.1010794.s004.tif]

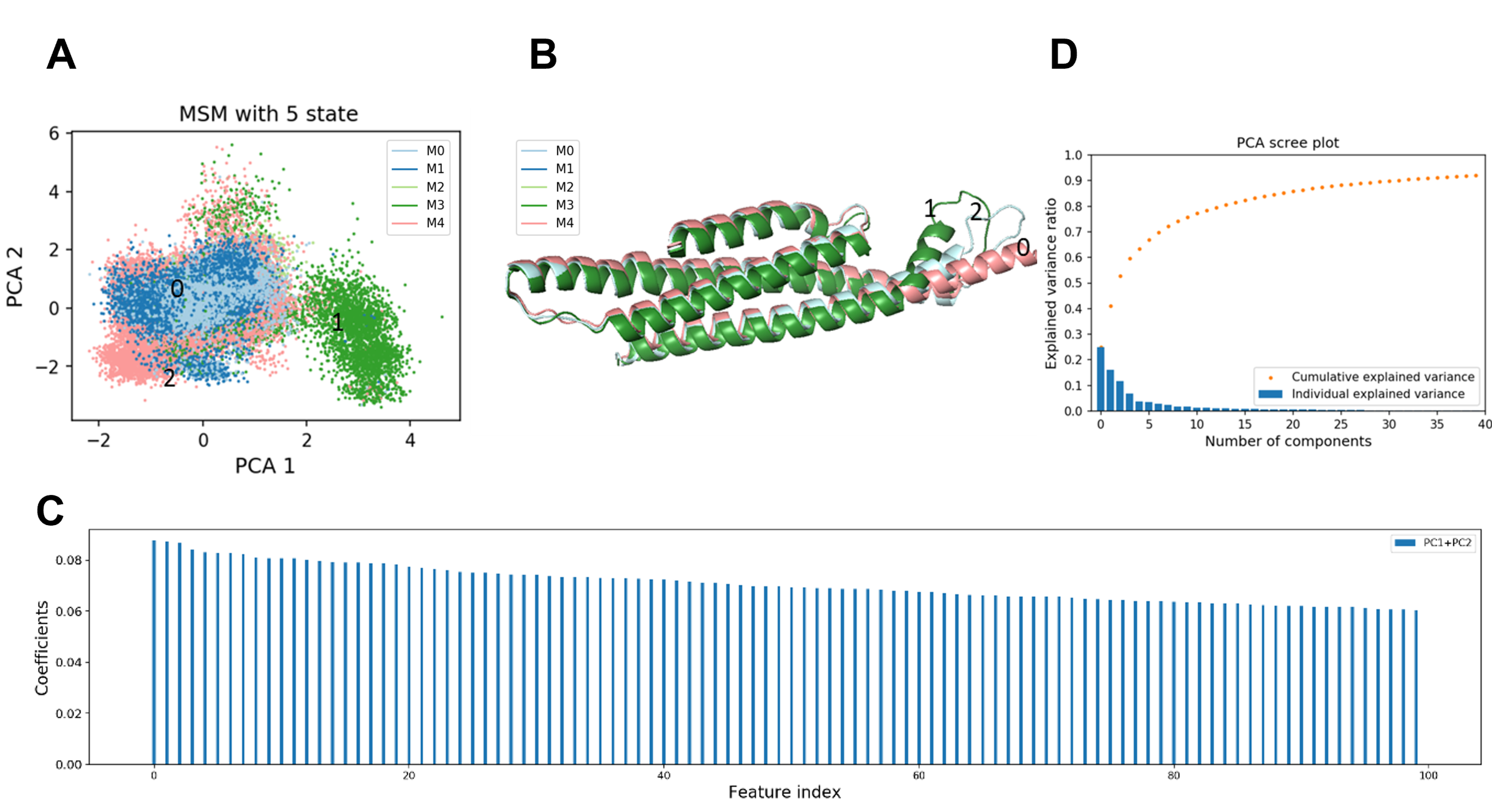

Supplement: S3 Fig — (A) PCA 2D plane of CCD pair Cα distances colored by macro-state from SH2 domain results. (B) Represent structure of CCD corresponding to Figure A. (C) The coefficients of first 100 features that contribute the first two PC the most. (TIF) [file pcbi.1010794.s005.tif]

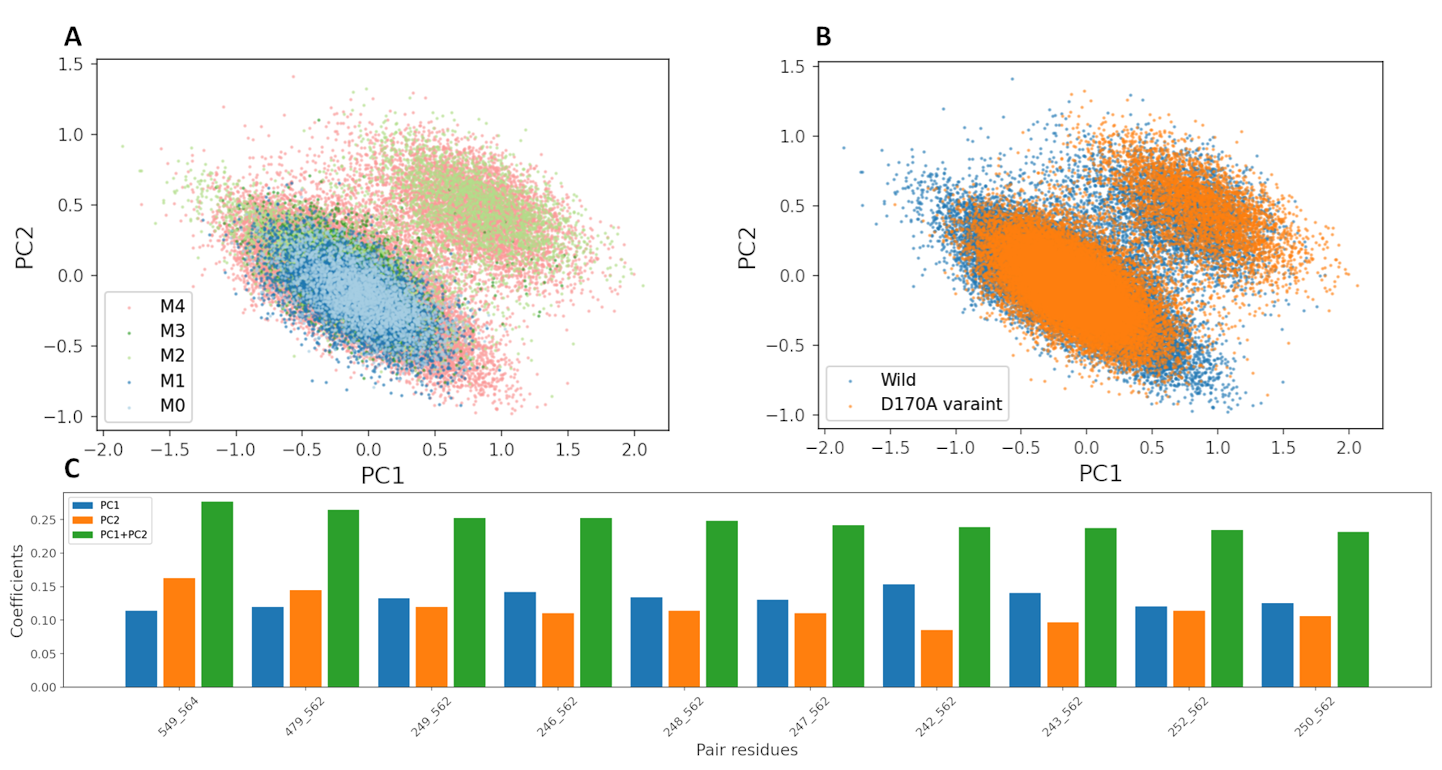

Supplement: S4 Fig — (A) PCA 2D plot colored by different macrostates; (B) PCA 2D plot colored by systems; (C) The first ten features that contribute the first two PC the most. The absolute value of PC1 and PC2, and square root of sum of squared PC1 and PC2 values are shown here. (TIF) [file pcbi.1010794.s006.tif]

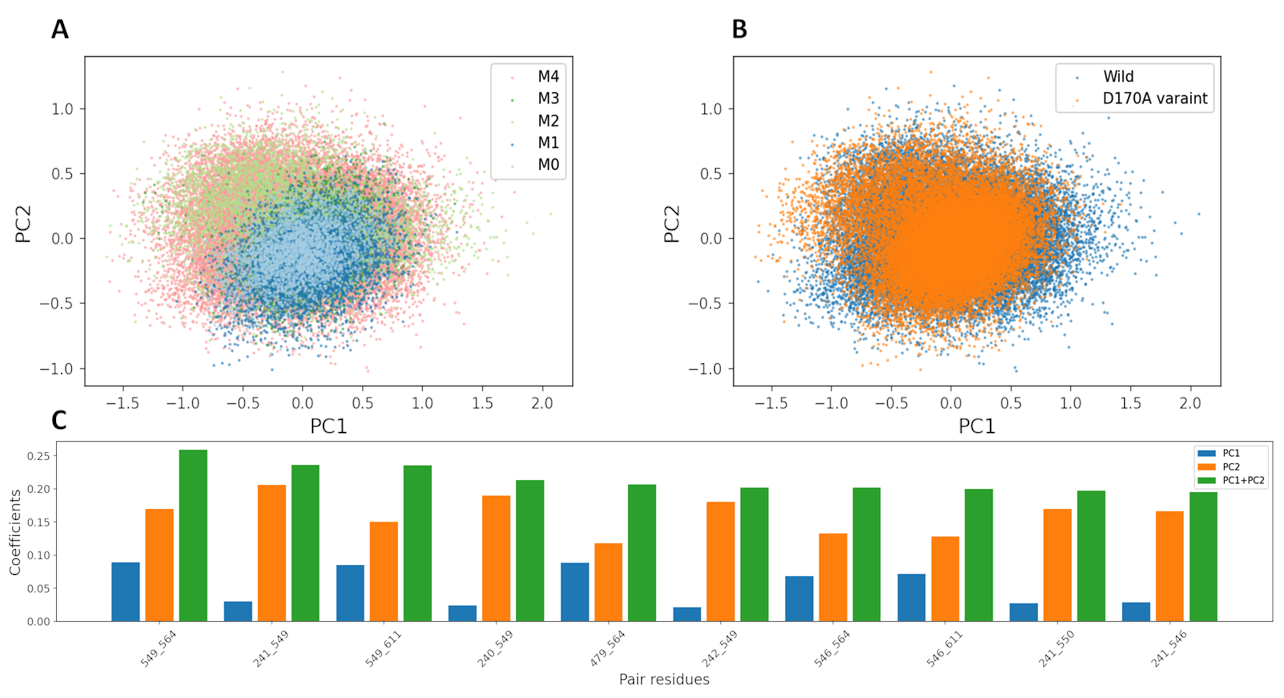

Supplement: S5 Fig — (A) PCA 2D plot colored by different macrostates; (B) PCA 2D plot colored by systems; (C) The first ten features that contribute the first two PC the most. The absolute value of PC1 and PC2, and square root of sum of squared PC1 and PC2 values are shown here. (TIF) [file pcbi.1010794.s007.tif]

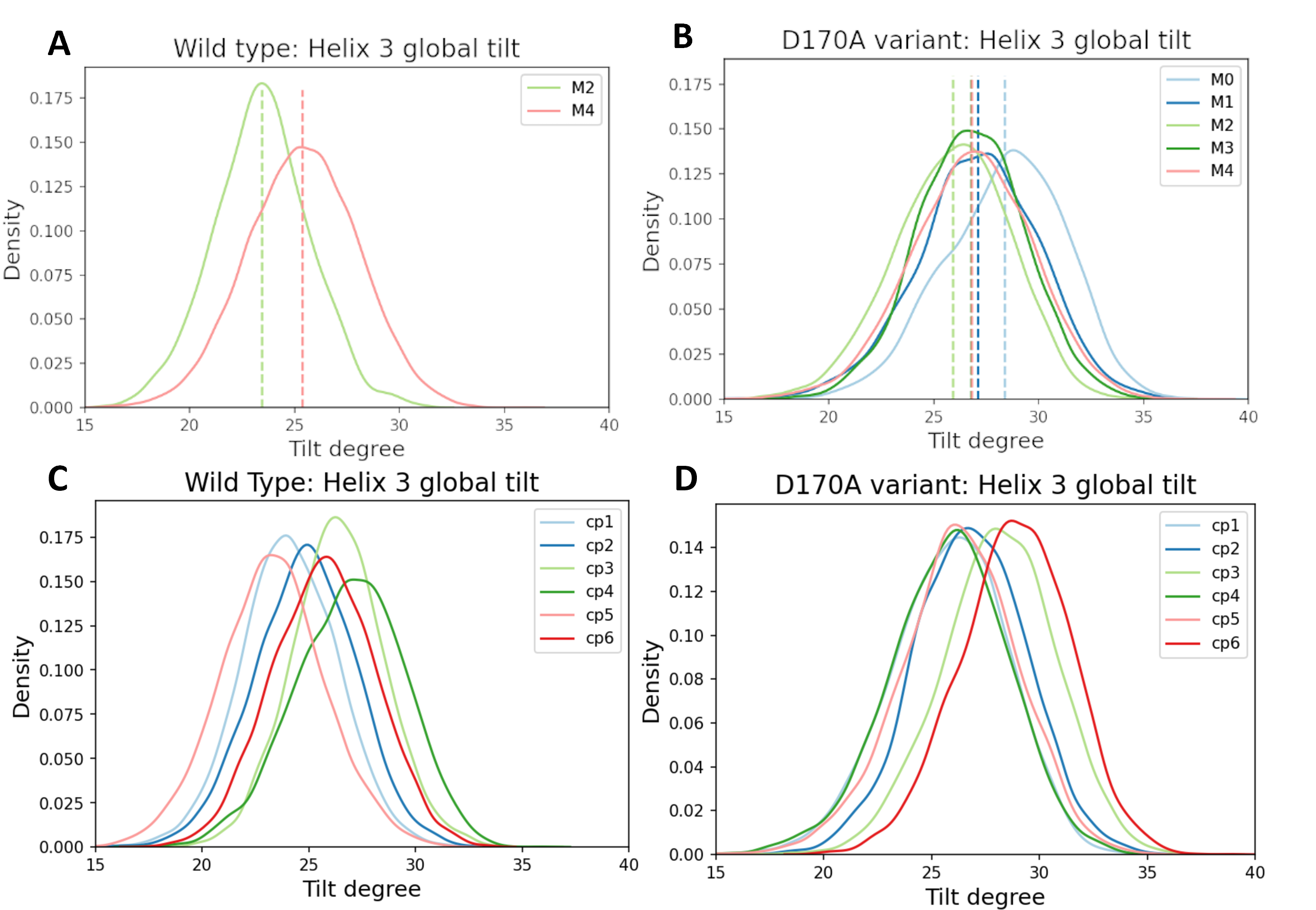

Supplement: S6 Fig — (A,B) CCD α3 global tilt angle distribution of different macrostates, plotted separately for the wild type and D170 variant. Average helix tilt angle within each macro-state is illustrated by a vertical dashed line. (C,D) CCD α3 global tilt angle distribution of different replicas for wild type and D170A variant. (TIF) [file pcbi.1010794.s008.tif]

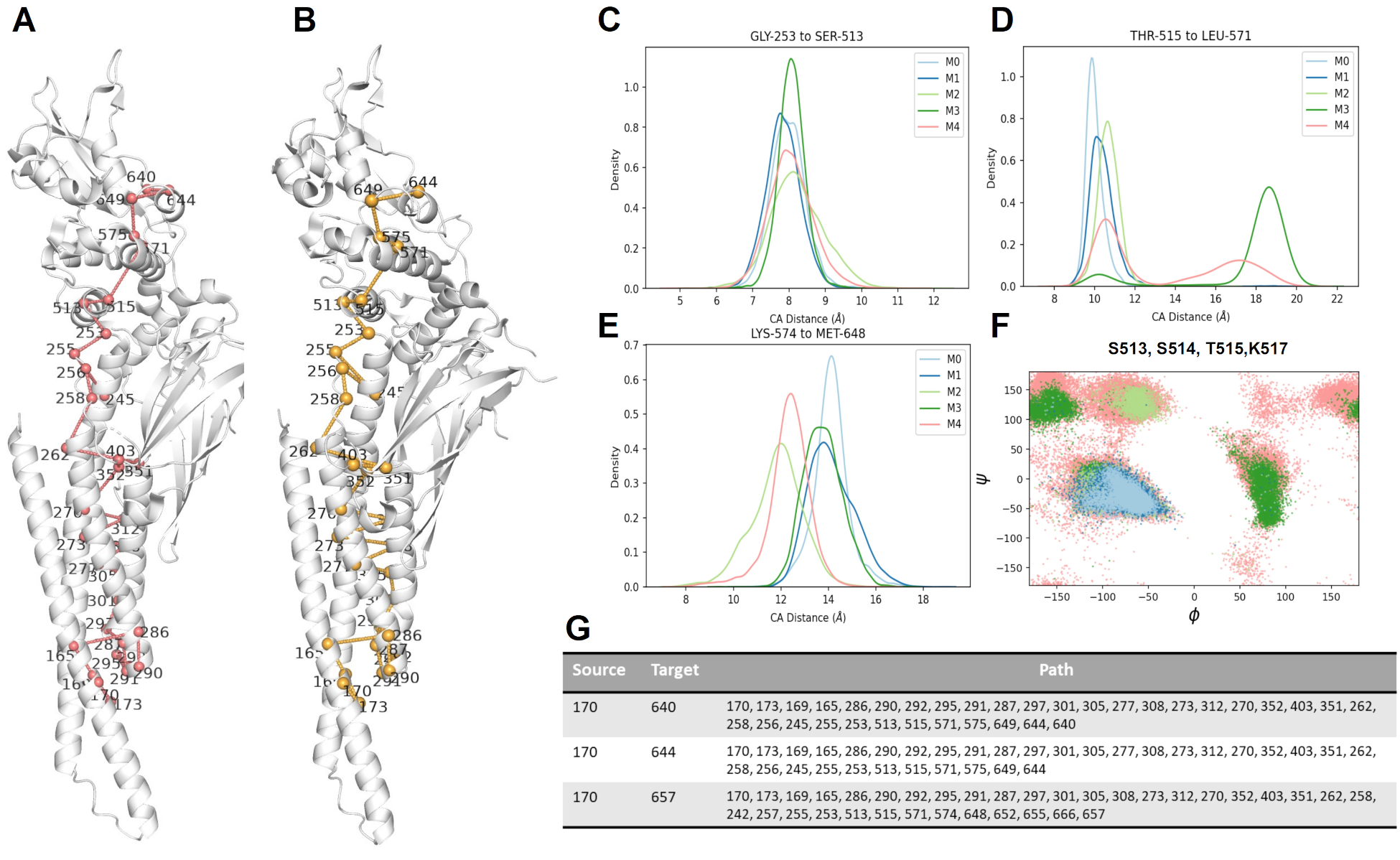

Supplement: S7 Fig — (A) Proposed pathway from 170 to 640 shown in the protein structure; (B) Proposed pathway from 170 to 644; (C,D,E) Key pair residue distance; (F) Ramachandran Dihedral for residue 513,514, 515 and 517; (G) Summary of proposed pathways from source residue 170 to target residue 640,644 and 657. (TIF) [file pcbi.1010794.s009.tif]

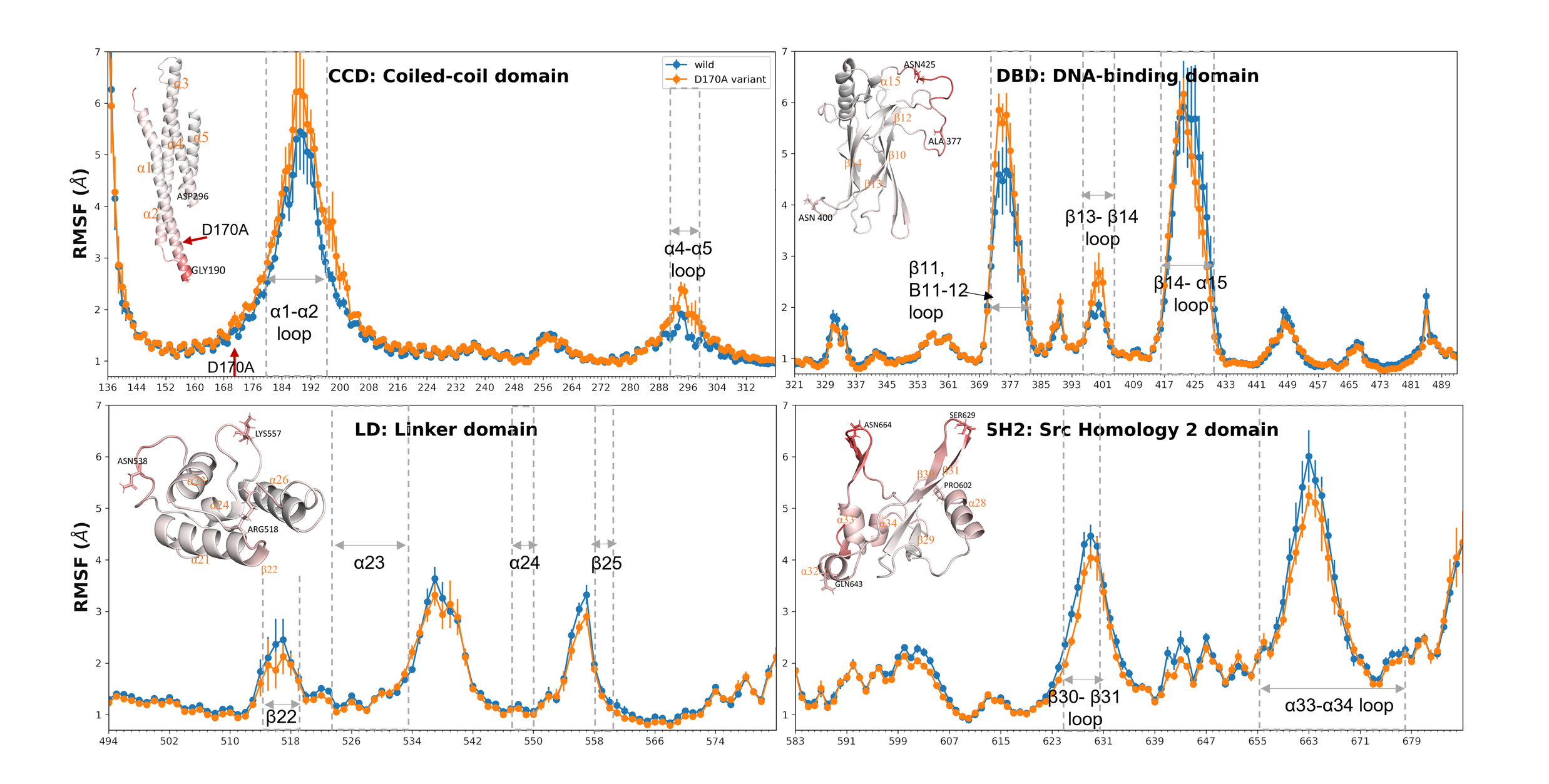

Supplement: S8 Fig — The wild type is plotted in blue and the D170A variant in orange, with error bar indicating the stand error among the six replicates. Structures of each domain colored by wild type RMSF values are shown (low RMSF values in white to high RMSF values in red). (TIF) [file pcbi.1010794.s010.tif]

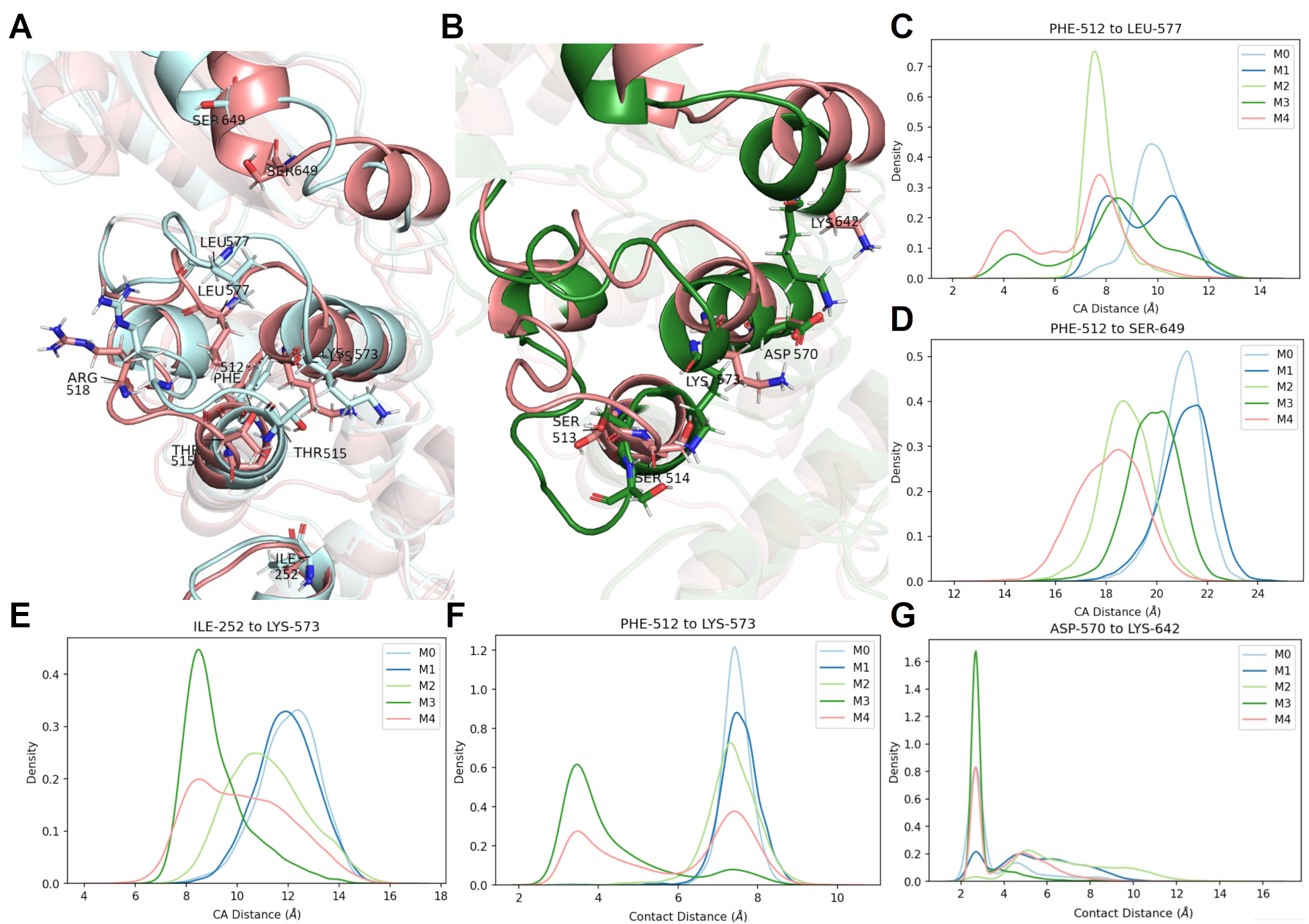

Supplement: S9 Fig — (A) Representative structure macro-state 0 (light cyan) compare with macro-state 4 (salmon); (B) Representative structure macro-state 3 (green) compare with macro-state 4 (salmon) (C-G) key residue pair CA distance or contact distance (closest heavy atom distance). ILE-252 to LYS-573 distance distribution and PHE-512 to LYS-573 distance distribution show α26 shift away β22 in macro-state 0 and 1, while toward in macro-state 3; PHE-512 to LEU577 and PHE-512 to SER-649 distance distribution show α26/α27 and α32/α33 loops move away β22 in macro-state 0, 1 and 3, while in macro-state 3, α32 moves close to α26 as indicated by ASP-570 to LYS-642 distance distribution. (TIF) [file pcbi.1010794.s011.tif]

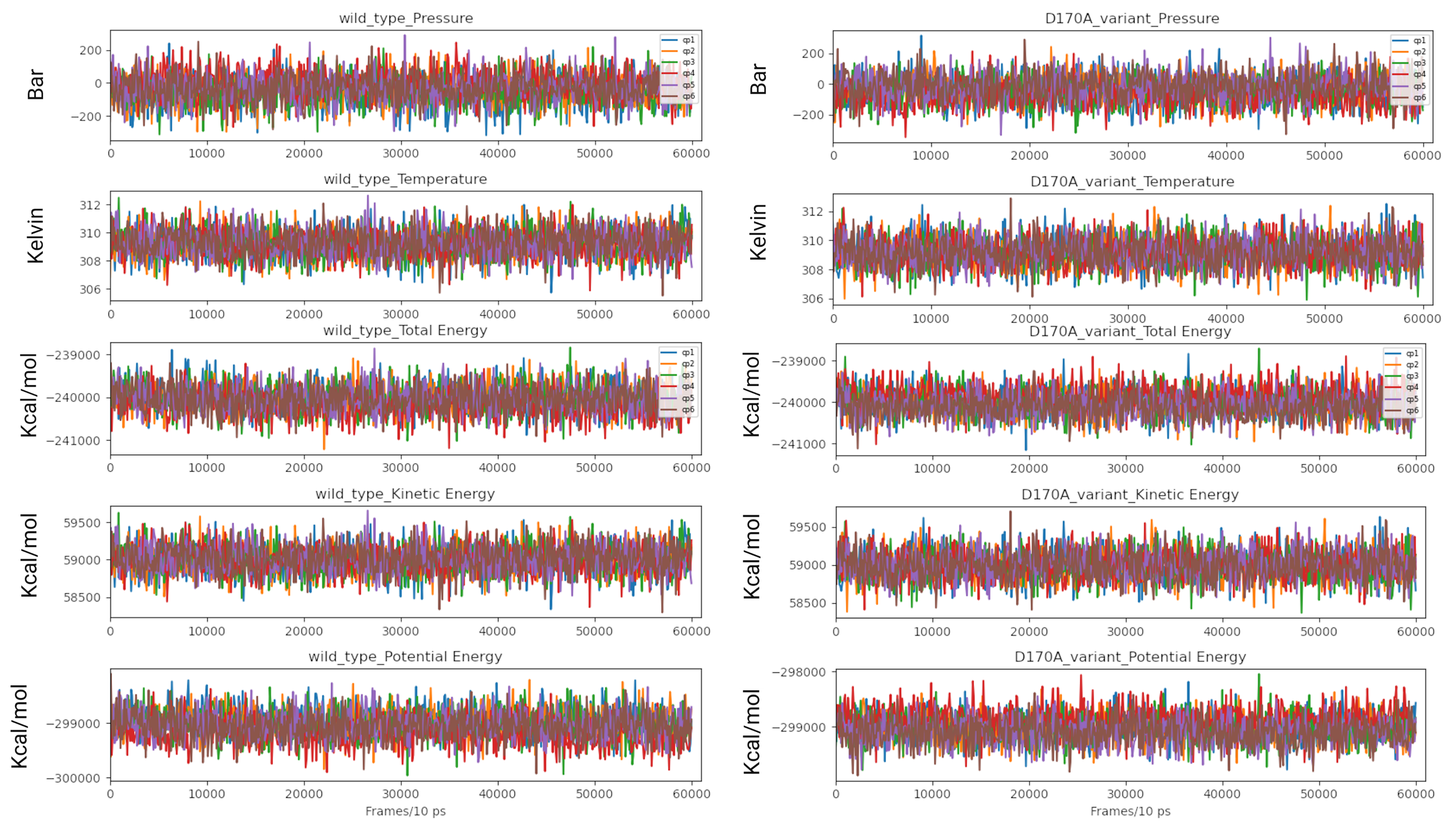

Supplement: S10 Fig — (TIF) [file pcbi.1010794.s012.tif]

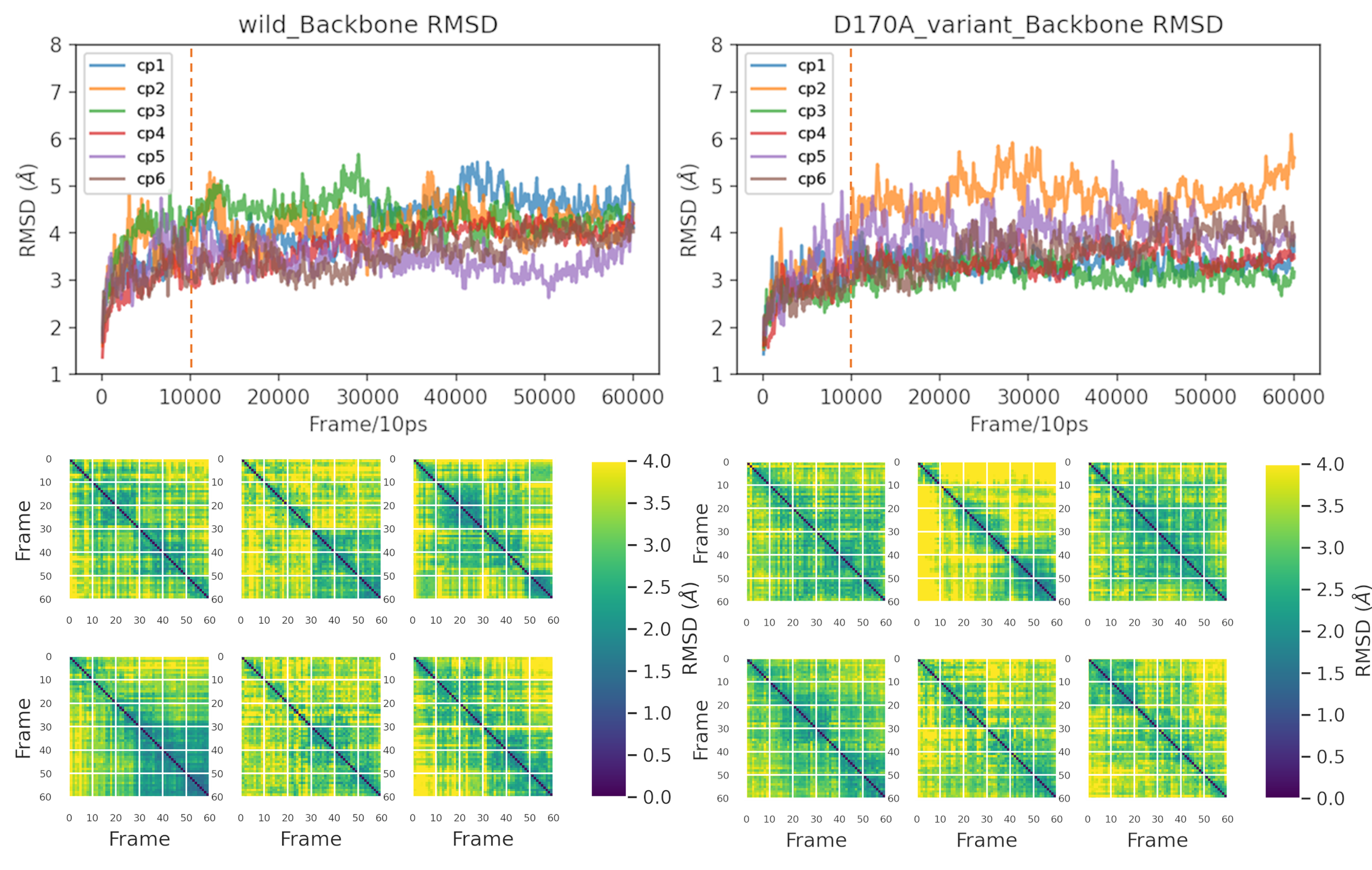

Supplement: S11 Fig — First row: Root Mean Square Deviation (RMSD) of wild system and D170A variant system, each system has six replicas: cp1, cp2, cp3, cp4, cp5 and cp6. Note: rolling average of every 100 frames was plotted here for better visualization. Second row: The pair RMSD (frames every 1 ns were extracted and used for pair RMSD calculation) of both systems for each replica. (TIF) [file pcbi.1010794.s013.tif]

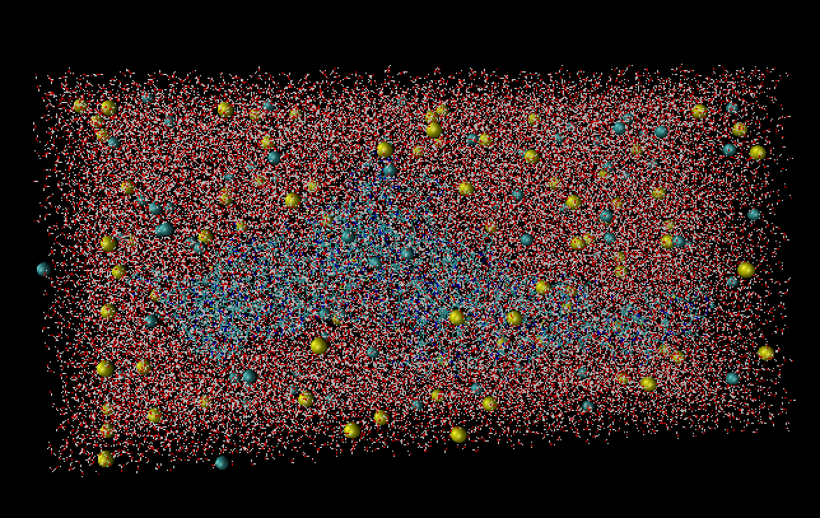

Supplement: S12 Fig — Water is shown as lines, ions are shown as vdw, protein is shown as cartoon. (TIF) [file pcbi.1010794.s014.tif]

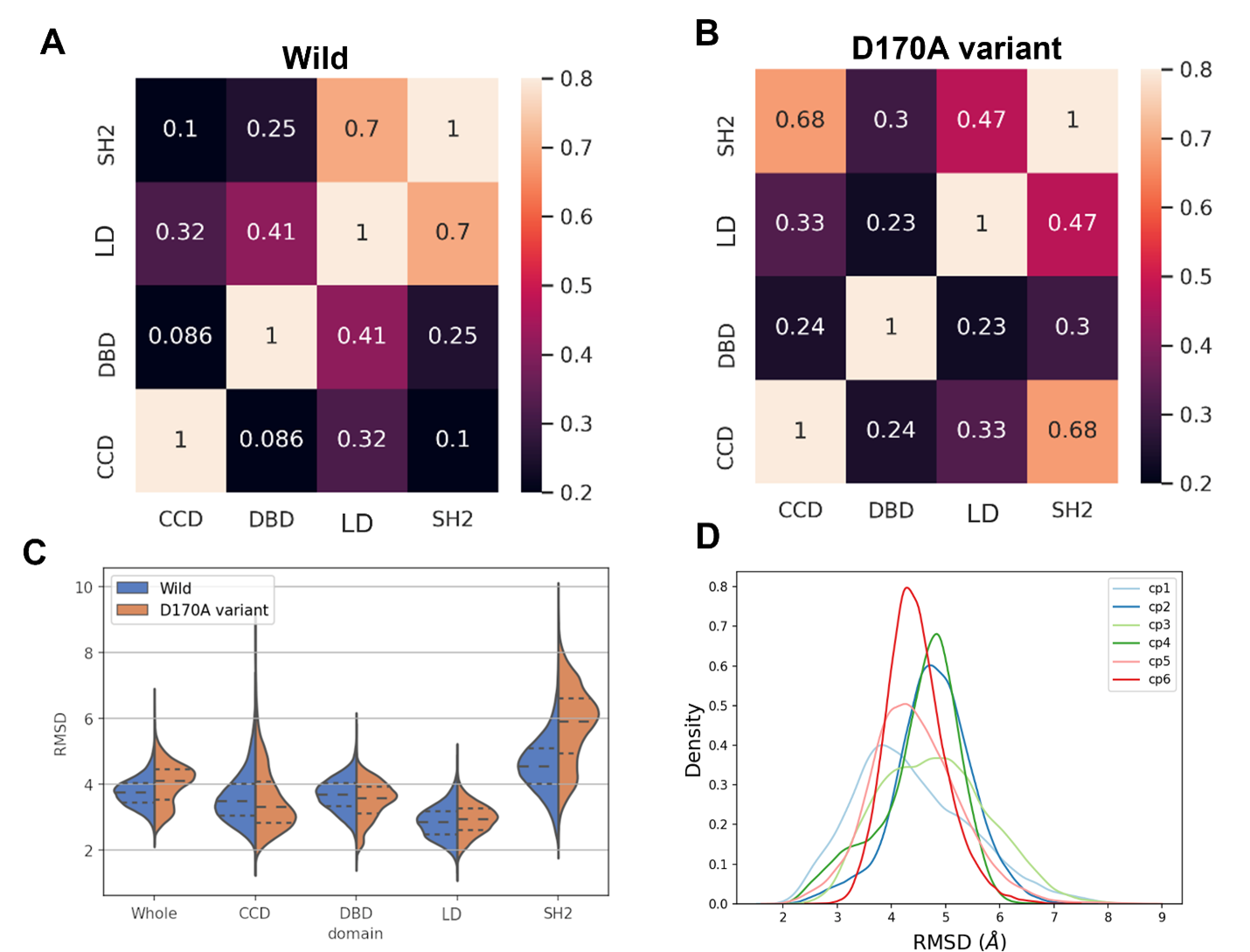

Supplement: S13 Fig — (A, B) Cross-correlation (Pearson correlation) of RMSD values of each domain for wild type and D170A variant. Here RMSD values were calculated with the first frame of reference since the correlation of dynamic changes of each domain is of interested. The cross-correlation was done by merging the rmsd of 6 copies trajectory together, and then the Pearson correlation among different domains were calculated. While worth being noted, the correlation value does not suggest the functional correlation between domains, since RMSD is an overall measurement of conformational changes with regarding to the reference structure, distinctive conformations may have same RMSD value. (C) Violin plot of RMSD values for the whole protein (core full length protein, not including NTD) and each domain. Here crystal structure was using as reference since the conformational changes difference between wild type and D170A was of interested. (D) The RMSD distribution of SH2 domain in the six replicas for D170A variant. (TIF) [file pcbi.1010794.s015.tif]
