# Supplementary material for: Allosteric regulation in STAT3 interdomains is mediated by a rigid core: SH2 domain regulation by CCD in D170A variant
Source: PLoS Comput Biol. 2022 Dec 21;18(12):e1010794. doi: 10.1371/journal.pcbi.1010794 (PMC9815575; doi:10.1371/journal.pcbi.1010794)
Supplement: Attachment — Submitted filename: response.docx [file pcbi.1010794.s016.docx]

Dear Prof. Gallo,

I would like to thank the reviewers for their thorough and constructive comments. We have carefully considered these comments and made the requisite changes in the manuscript (a separate version of the manuscript with changes highlighted in red has been provided for convenience). In addition, our detailed responses to the reviewers’ concerns and queries are included below. We hope that the manuscript is now ready for publication in PLOS Computational Biology.

Sincerely,

Devin Matthews

[damatthews@smu.edu](mailto:damatthews@smu.edu)

**Reviewer #1**

While I do not feel qualified to comment on the importance of understanding STAT3, or the current knowledge regarding this protein, I am qualified to evaluate the molecular dynamics simulations and analysis. I found this manuscript to be engaging, and I believe that this study has high scientific rigor. The authors were very diligent and careful in their simulations and analyses.

My primary criticisms, as you will find detailed below, primarily deal with the clarity of the take home message. I think there are things that should be removed from the paper because they confound the story. Also, some effort should be put into clarifying figures and text.

**Significant Suggestions:**

1. While this is a significant modification, it will be an easy suggestion to implement. In the Methods section, there is no need to give equations or elaborate on details that are taken care of by the software. For example, for RMSD, just tell the reader what the RMSD measures in a sentence or two, and then tell them the software (MDtraj) and the command(s) you ran (presumably mdtraj.rmsd). If there are cases where you actually generated values and then plugged them into formulas, then tell us, but if it’s baked in the software then why bother.

Thanks for your suggestions, the methods have been simplified according to the suggestions and now more accurately reflect the calculations done directly by the authors.

2. An overall suggestion that encompasses several points below is to ask the following question for each figure/subsection: Does this support and clarify the take home message by adding a new point, or is it unnecessary? If it is unnecessary, then don’t include it in the manuscript. I think in some cases (e.g., perhaps RMSD, RMSF), these analyses led you to the more advanced (e.g., MSM). However, if they don’t add anything to the story, then it would be better to include them in a supplement than the main text. Otherwise, it confounds the main story and readers are left trying to understand what they are supposed to learn from a figure. A specific example of this is the RMSD analysis in the paper. I certainly do understand why you would have done this analysis as part of the study, but it doesn’t appear to add any value to the story that isn’t more clearly illustrated later. Especially given the point you make yourself in the caption, that different conformations can have the same RSMD. I would suggest removing this or moving it to a supplement.

Thanks for the suggestion. After revisiting the manuscript, we have decided to move the RMSD/RMSF result figures to the supplemental material. While we do still think that the results add important information and support our conclusions, the figures aren’t necessary in the main text.

3. In fig 3 you chose to use the deepness of the color to represent standard deviation. I would suggest two modifications to this choice. (1) Use error bars rather than color depth. This allows the reader to easily assess the significance of the data points where there is a difference between the mutant and wt. (2) Show standard error rather than standard deviation (i.e., stddev/sqrt(6)). Again, this better allows us to evaluate the significance of the differences. The standard error quantifies the precision of your RMSF values rather than the spread of the RMSF values in individual runs. Also, some more minor points regarding fig 3. Please show the location of the mutation site. The whole point of the figure is comparing wt and mutant, yet I can’t tell where the mutation occurs from your figure. In the caption I assume “stand deviation” is meant to read “standard deviation”. Also, some font sizes are too small. Increase fonts sizes for axis labels, domain/site labels and the key in the first panel. Finally, I find the domain labeling to be confusing. What does \alpha1-\alpha2 mean? Why not just put the \alpha1 label within the dashed lines that represent the boundaries for that sub-domain?

As discussed below, the different replicas explore distinct areas of configuration space (e.g. they sample distinct quasi-stable configurations). Thus, the reason for illustrating the standard deviation between replicas is to give an idea of how the flexibility of the structure changes between these configurations. We ultimately arrived at this representation (with depth of color indicating higher stddev) after trying other strategies, including separate RMSF curves, error bars, etc. and finding that the present approach was the cleanest and, to us, the most informative. We switched back to error bars upon moving the figure (updated to Figure S4) to the SI. Other aspects of the figure have been improved as indicated.

4. In fig 4 there are some hard to read fonts. In particular, the labels in panel D, G are hard to read. The yellow and orange pY and pY+3 labels could perhaps be outlined in black to enhance readability. All labels in these panels should use larger (and perhaps bold?) fonts. Also, it is not clear why you compared to 6nuq here when you were using a different PDB structure up to this point.

Thanks for the suggestions, Figure 4 (updated to Figure 2) was modified to enhance readability.

6NUQ and 6NJS are the two pdb structures with the ligand binding at SH2 domain. Only 6NUQ is shown in the plot as both the structures bind the same ligand and are highly similar. It is used here as a reference of the binding conformation. 6TLC was initially selected as model protein to set up the simulation as there are no missing residues in CCD. We added a note to the Figure 4 (updated to Figure 2) caption to reflect this.

“6NUQ is STAT3 with a ligand bound to the SH2 domain, shown as a reference for the ligand binding conformation.”

5. I found fig 5 to be very confusing. On page 13 you state “The existence of such an interaction network is demonstrated in Figure 5B, where the inter-domain pair Cα distances are plotted and colored according to standard deviation values computed across all trajectories.” I think you mean 5A here, but it still doesn’t make sense to me.

For each plot you show 5 curves (M0-M4) and they appear to be color coded by this, not the standard deviation. M0-M4 is not defined anywhere. Reading ahead it looks like you might be referring to the macrostate number here? Anyway, the figure caption and manuscript text need significant clarification.

A couple more minor points, I would suggest changing your language regarding “hydrogen bonds distance”. I think what you mean to say is distances between atoms that form hydrogen bonds. The reason for this change is that your distributions reach well past what most people would consider to still be a hydrogen bond, i.e., the bonds appear to be breaking sometimes. Finally, there are again some very small fonts. In panel B, there is a sub-plot that is quite literally unreadable at any zoom level. As before, consider both font sizes and colors.

We apologize for the confusion. The figure legend has been updated for clarity and the labels in Figure 5B (updated to Figure 3B) sub-plot was enlarged. Specifically, we state that the analysis is done on various macrostates and add a supplement Figure S8 for helical tilt on the wild-type and D170A variant. We changed the wording from hydrogen bond distance to the “Distance distributions of the atom pair, that form hydrogen bonds” in order to address any type of interaction and distance regime.

6. The analyses presented in fig 5 and fig 7 seem to have a lot of overlap. While fig 5 includes some rigid core analysis, the hydrogen bonding part has a similar take home message (but is less clear to me) as fig 7. It is not clear why both are needed. I’m not saying you should eliminate fig 5, but perhaps consider removing the parts/panels that are redundant.

We apologize about the confusion; however, we argue that there is no overlap between figure 5 and 7 (updated to Figure 3 and 5). Although both figures concern hydrogen bonds (at least in part), Figure 5 shows commonality between various macrostates (conserved interactions forming the rigid core) while Figure 7 shows the differences between M0, 1, 3 and 4 which lead to translation of movement and allostery. The caption for both Figure 5 and Figure 7 has been updated to enhance clarity.

7. It is mentioned that MSM analysis was performed. Fig 4 shows some clustering results, but there is no mention of the transition probabilities in the main text. Does this analysis not add anything to the story?

While we have computed transition probabilities between different states as part of MSM based clustering, we hesitate to add meaning to the transition probabilities due to inherent limitations of MD simulation and the nature of the clustering process. The Perron cluster-cluster analysis (PCCA) that was used for coarse graining the MSM states has a known limitations on poorly sampled transitions. MD simulations is optimized to sample all conformational space provided that very long simulations are performed with large number of replicates. The computational cost to show exhaustive sampling of conformational landscape compared to the kinetic information obtained was deemed to be infeasible. However, the rough kinetic model obtained from the present data is sufficient to define kinetically relevant clusters for further analysis.

**Minor points:**

1. Page 2: “amino-terminal domain ()”, should there be something in the parentheses?

Yes, it is. It has been added.

2. Fig 1 caption: NTD not defined, perhaps this ties into the first minor point?

Yes, it is. It has now been appropriately defined.

3. Fig 1: Consider showing the location of the mutation site D170 that you are studying. I ended up figuring it out from going to the PDB because I wanted to know when looking at this figure.

We have added a label in figure 1.

4. Page 6: “RMSD stabilized around 4”. Without looking at the figure I would assume angstroms, but you need units here.

This has been added.

5. Fig 6: Residue labels in panel A are hard to read. Making them bold might help, but part of the issue is that some are very crowded and some are obscured by the dark blue line.

The number of residues identified by REDAN and their position makes it difficult to visualize the labels. PyMol session files (pse, available at https://osf.io/dvzq7/) are uploaded as supplemental material with the labels has been uploaded for visualization by the readers. Furthermore, to address similar issue, pse file is made available for the rigid core analysis as well.

**Reviewer #2**

In the paper “Allosteric Regulation in STAT3 Interdomains is Mediated by a Rigid Core: SH2 Domain Regulation by CCD in D170A Variant”, Davin Matthews and co-workers analyze by means of Molecular Dynamics simulations the effect of the D170A changing on the dynamics of the STAT3 protein. In the paper, they put in evidence that the two variants of the protein populate a different conformational space. In particular, a different conformational state of the SH2 domain. Overall, the reported data seems to be convincing, however, in my opinion the helices in the Coiled-Coil domain populated in the D170A variant affects the dynamics of the pY+3 binding site in, in some cases, the statistical relevance of the reported data needs to be improved to prove that the discussed picture is not a consequence of stochastical fluctuations of the simulated systems.

1. The authors acquire six different independent trajectories for each variant; inter-trajectories variability could be inserted to make the discussion more convincing.

We have added a paragraph to the Discussion which motivates and validates the use of six independent trajectories. The (largely distinct) conformational spaces explored by these trajectories are closely related to the macro-states uncovered by our kinetic clustering analysis, and together effectively sample the global conformational dynamics. As explained in the new text, the 2D PCA coverage shows that the relevant configurations for SH2 structural differences are well-sampled. Of course, the reviewer is correct that inter-trajectory variability is still an important piece of information (alternatively, we could have used the standard deviation of representative macro-state geometries, etc.). This information is included in the SI as error bars on the RMSF analysis(Figure S4).

“The six independent trajectories obtained for each variant explore fairly distinct areas of conformational space (i.e. different macro-states as identified in the clustering analysis, see Figure 2H). The long transition timescale between these states validates our kinetic clustering model, but also necessitates a “global” view of the trajectories in order to explain the full conformational dynamics of the protein. Based on the coverage of the 2D PCA space of the SH2 domain (Figure 2), we find that the six trajectories obtained are sufficient to sample the dynamics relevant to ligand binding affinity. On the other hand, the long transition timescale necessarily leads to high variability between trajectories for certain computed properties such as RMSF (Figure S4). While not an indication of insufficiency in the global conformational view obtained from combining all trajectories, this variability instead serves as a valuable additional measure of conformational differences between kinetic macro-states.”

2. Another weakness of the paper that could be strengthened is the lacking of a discussion of a clear effect of the mutation on the interaction with its surrounding residues. One of the conclusions of the authors is that more than a different induced flexibility, the change in the residue 170 acts through a “rigid core mechanism”. This mechanism is described by proposing a clear sequence of interactions between the coiled-coil and the SH2 domains. The table in figure S4G clearly indicates this sequence of interactions, but, if this is the case, a clear different picture in the nearest-neighbor interactions of the residue 170 in the wild-type protein and in its D170A variant should be observed. Quite surprisingly, the authors do not search for these differences; I think that these data could contribute to making the hypothesis of the authors more convincing.

We have added a paragraph to the Discussion which motivates our choice to leave out the local interactions and perturbations in CCD. Essentially, we are looking towards a unified explanation of allostery for a variety of effectors targeting CCD. While we only explore D170A in this work, we felt that an over-emphasis on CCD itself would be counter-productive given the likely differences in the initial pathway leading to the critical CCD-LD interactions (esp. the CCD helix tilt angle). In addition to the new paragraph in the Discussion, we have added the following text to the Introduction in order to motivate our choice of D170A alone for this investigation.

“There are no crystal structures with MM-206 or K116 binding with STAT3. Moreover, computational modeling of large fragments and optimizing the accurate binding configuration is challenging. D170A mutation which has shown altered activity in SH2 domain can, thus, be useful avenue of investigation towards understanding the allosteric mechanism. This has further advantage in that a single point mutation can easily be performed computationally without significantly altering the structural environment.”

Of course, a full investigation of D170A in combination with these other effectors is the desired end goal, but is beyond the scope of a single manuscript.

3. Finally, my last major concern regards the linking to the experimentally observed effect of the D170A changing. What is experimentally observed is a lowering in the affinity of mutants with respect to the wild type. What the authors observe in their dynamics is higher flexibility in the pY+3 binding pocket in the D170A variant. The authors would to better discuss this aspect, for example by showing that in similar systems a correlation between these so different observables has been demonstrated.

The link between binding pocket and lowered binding affinity and has been explored in the following papers (among others):

<https://doi.org/10.1073/pnas.0610064104>

<https://doi.org/10.1371/journal.pcbi.1003782>

These references have been added to the text and the concept is introduced using following amendments:

“These observations highlight CCD correlation to SH2 domain binding affinity as well as specificity.”

“In biomolecular recognition, the two aspects binding affinity and binding specificity are coupled to each other, i.e. strong binding affinity is indicative of high substrate specificity and vice versa. Furthermore, strong coupling among flexibility and binding affinity has been shown in different systems [https://doi.org/10.1371/journal.pcbi.1003782; https://doi.org/10.1073/pnas.0610064104]. Thus, higher flexibility would lead to exploration of extended conformational landscape and reduced occupancy in substrate binding conformation. The changes in flexibility of pY+3 pocket could potentially explain the reduction of binding affinity observed by Zhang et. al.”

Here below, I report minor concerns/comments:

1. The paper needs an editing revision. For example:
   a. at page 3, in the first lines of the introduction section, the acronym for amino-terminal domain is lacking;
   b. in the caption of table s1, uniport instead than uniprot is reported;
   c. at page 12, figure 5H does not exist, probably the authors refer to figure 5F;
   d. In the methods section (in the first paragraph) the reported pdb code is wrong (6tle instead than 6tlc);
   e. at page 24, “is calculated” is repeated two times

Thanks for the kindly pointing these errors out; they have been fixed.

2. In figure 1B, residues clearly folded in helical structures are colored in yellow, a color that indicates unstructured regions (loops) according to the caption. Is the structure wrong or the problem is in the assignment?

The secondary structure assignment was obtained from the UniProt database. The database reports that these assignments are compiled from multiple x-ray crystal structures. The x-ray crystal structure shown in the figure, however, has a helical fold. In fact, these regions dynamically change their fold during the simulation. An explanatory statement has been added to the caption.

“The assigned secondary structures combine information from multiple x-ray crystal structures, thus there are some mis-matches with the specific structures used in this work.”

3. At page 4, in the description of the SH2 domain family, the authors mention the pY+3 interactions a as specificity region. This is probably the case for the SH2 domain in STAT3, but, this statement is far to be general, and wider regions (from -2 to +5) can be determinants for the selectivity (see for example the case of the N-SH2 domain in the SHP2 phosphatase). More in general, the discussion at page 8 seems to develop on two different planes, that of the SH2 domain in STAT3 and that of the SH2 domain in general. The authors could be more clear, defining each time at which SH2 they refer.

Thanks for pointing it out, this clarification has been added to the main text.

4. At page 4. The STAT3 regulation mechanism mainly discussed in the paper is that the phosphorylation of Y705 promotes STAT3 dimerization, by binding of the pY705 in the SH2 domain of another STAT3 protein. The general motif for the SH2 domain in STAT3 is described as pTyr-Xaa-Yaa-Gln. Quite strangely, the sequence surrounding Y705 (SAAPpYLKTKF) does not contain this motif. This point resembles my previous comment on the effect of mutation on the binding affinity. A change in the pY+3 binding pocket could reflect changes in selectivity more than in affinity. Commenting on this aspect could help the discussion.

Thank you for the insightful question, gave us the opportunity to rephrase the take home message of our study for better clarity and avoid misleading conclusion. pTyr-Xaa-Yaa-Gln is a motif with higher binding affinity due to the additional interaction of Gln with pY+3 pocket, and the unique interaction in pY+3 pocket also increases the specificity compared to STAT1. However, for its function, pSTAT3 needs to dimerize and thus the sequence around Y705 could bind differently compared to the typical pTyr-Xaa-Yaa-Gln motif. As such, we have added discussion to describe that the pY+3 motif is more dynamic and concluded that inhibition at both stage of STAT3 function: phosphorylation and dimerization may be impacted.

“In short, the mechanism for signal transduction has been identified through analysis of dynamic correlated motions between CCD and SH2 domain, the exact outcome of this allosteric effect is not clear. Distinguishing between two potential mechanisms: changes in substrate specificity to form heterodimer required for phosphorylation of Y705 and reduction in homodimerization due to reduced affinity towards pSTAT3, needs to be investigated.”

5. - a brief discussion on the biological interest (if any) for the D170A variant could be of interest (e.g., is this variant causative of pathologies?).

D170 is an artificial mutant, and was chosen based on the reported systematic deletion analysis of the N-domain and α helices of CCD which were carried out to study the communication between CCD and SH2 domain, as well as mutagenesis of conserved residues (D170A) in the CCD of STAT3 showed the diminishment of both pY-peptide binding and tyrosine phosphorylation. Additional text has been added to introduction to reflect our specific interest in D170A.

“There are no crystal structures with MM-206 or K116 binding with STAT3. Moreover, computational modeling of large fragments and optimizing the accurate binding configuration is challenging. D170A mutation which has shown altered activity in SH2 domain can, thus, be useful avenue of investigation towards understanding the allosteric mechanism. This has further advantage in that a single point mutation can easily be performed computationally without significantly altering the structural environment.”

6. - The authors use the RMSD from a single structure to prove that the system is well-equilibrated. This approach could be misleading (see for example: J. Chem. Phys. 1998, 109:10115 or Comput Math Methods Med. 2012; 2012:173521). Flat (sometimes high) values of the RMSD reflect only that the sampled conformations are all equally distant from the reference but little can be said about the distance between them. RMSD computed by comparing all the frames to each other (with results reported as a graphical matrix) could be more reliable. In this context, quite surprisingly, the RMSD values seem to be higher for the SH2 domain in the wild-type simulations than in those of the mutant. How do the authors comment on this evidence?

We would like to thank the reviewer for pointing out our misleading use of the term well-equilibrated. As such we have referred to the papers suggested by the reviewer to reconcile between what we intend to portray and the actual portrayal of the information. The two papers referred by the reviewer demonstrates the convergence of the MD simulation to a local minimum. We have added a heatmap to show that our trajectory is not stuck in a local minimum as an artifact of minimization and heat up protocols. We’ve added text in the article to elaborate on our use of RMSD as a measure of conformational sampling of our MD simulation. Specifically:

“Sufficient sampling of the MD simulation for both wild type and D170A variants was evaluated by the pair Root Mean Square Root (RMSD) (Figure S2), which shows that the simulations have either reached a stationary shape or there is transition between different stationary shapes.”

D170A variant

Wild type

7. - Some regions of the protein have been modeled because absent in the pdb file. In the scheme below the figure 1A (in which the different domains are indicated) the modeled regions could be somehow put in evidence. This information is practically absent in the paper, but it can help to interpret the RMSF data.

Thanks for the suggestion, this information has been added in the method section.

“In chain A, residue 372-381 and 418-428 in DBD domain were missing, here these residues were modeled using Chimera”

8. - At page 7, the statement " D170A variant has multiple quasi-stable conformations seen as multiple peaks in the core full length protein violin plot" in the comment to figure 2c needs further comments.

The time evolution of RMSD (with respect to a different structure) reported in figure S1 seems to suggest that this behavior is not homogeneous on the six replicas. Probably the RMSD distribution in the six replicas could be helpful to understand if only a few simulations are responsible for these differences or if they are observed in all the simulations

We appreciate the feedback on our claim of multiple quasi-stable conformations. We further investigated the proposition by using suggested analysis on all the replicas. We have added the figure in Figure S3D and text in the supplement:

“The RMSD distribution of SH2 domain in the six replicas for D170A variant were also plotted (Figure S3D), the multiple quasi-stable conformations in Figure S3C were observed in a few simulations, with the others explore one stable conformation, which is consistent with the observation form pair RMSD plots that the simulations have either equilibrated into a local minimum or transition between local minima.”

9. - At page 12 "While the rigidity of the α helices diminished the utility of PCA analysis, it also demonstrates that the dynamics of the helices are highly correlated (Figure 3)." How does figure 3 demonstrate the correlations between the dynamics of the helices?

As per the reviewer’s suggestion, we amended the description in the text to state the correlation being present in literature and how we generated a testable hypothesis to study the phenomenon. Reference to Figure 3 has been removed and the following text has been updated:

“The rigidity of the α helices diminished the utility of PCA analysis, however the communication between CCD and SH2 domains have been observed in the literature. To investigate this correlation, we hypothesize that the long helical arms of CCD may act as rigid levers, where a slight helical tilt causes significant changes in inter-domain interaction sites.”

10. data in figure 2 are acquired from six different simulations. How much is the standard deviation between the different simulations? These data should be added to figures 2A and 2B. For example, the reliability of the statement "This correlation is enhanced at the expense of correlation of different domains to the linker domain, with only CCD retaining its correlations with linker in D170A variant." depends on the values of the standard deviations.

As we noted above, the use of the aggregate of all six replicas is the most relevant approach for the bulk of our analysis. However, the inter-replica variance is illustrated in the RMSF data (Figure S4) and other places (e.g. Figure S3D).

11. in a comment on the RMSD data the authors write " Overall, the deviation of the SH2 domain from the crystal structure is significantly increased and several quasi-stable conformations appear, while the CCD domain becomes somewhat more rigid." RMSD takes into account differences between structures, and much less about flexibility. Under this point of view, RMSF is definitely better. By the way, the RMSF values seem to suggest the opposite behavior.

We reworded this statement to better reflect our intended message. RMSD shows differences in structures as pointed out by the reviewer, which is important in context of comparing conformational characteristics. However, the comment on rigidity should be reserved for RMSF analysis as the dynamical changes is more clearly described by RMSF. We intended to comment on the conformational landscape and have modified it to reflect as such.

“Overall, the deviation of the SH2 domain from the crystal structure is significantly increased and several quasi-stable conformations appear, while the CCD domain explore similar landscape with crystal structure.”

12. - by looking at figure S2, the distance between 644 - 657 and 638-644 can be used as an approximate representation of PC1 and PC2 (as also shown in figures 4 B, C, E, and F). The plot of these two distances in the 12 simulations could help to understand how the PC1-PC2 space is populated in the different simulations.

Thanks for the suggestion. As we noted above, we have added text to highlight our reasoning for analyzing and presenting aggregated data only. And the two distances of both wild type and D170A variant for each replica of simulation have been plotted. This figure (Figure S11) has been put in supplement for transparency of the variation in the inter-trajectories.

13. - the sentence " The motion of Y657 and Q644 yields the largest coefficients across both PC1 and PC2, underlining their conformational importance" at page 9 is misleading. The authors refer to the addition of the two contributions to PC1 and PC2 but the distance contributes only marginally to PC2.

Thanks for pointing out this confusing claim. We intend to express that “The motion of Y657 and Q644 yields the largest coefficients across PC1 and PC2, respectively, underlining their conformational importance.” The change has been made in the main text.

As shown in the following figure, that PC1 mostly reflect the variance of relative position of residues to residue Y657, while PC2 mostly reflects that to residue Q644. The original figure 4A has been replaced by the figure below.

14. - How much of the explained variance is described by PC1 and PC2 vectors in the PCA analysis reported in figure S3?

The explained variances that described by PC1 and PC2 vector are around 0.25 and 0.16, respectively. The following figure has been added to supplement Figure S6.

15. - It is not clear if the data reported in figure 5 refer to WT, to the D17A variant, or both of them.

Thanks for the kindly pointing out, the data reported refer to both systems combined, and the information has been added in the caption of Figure 5(updated to Figure 3).

“The analysis includes all trajectories spanning macro-states M0–M4.”

16. - figure 5B is hard to read. A table reporting the percentage of existence for all the considered interactions would be more useful

Sorry about that. The labels of Figure 5B (updated to Figure 3B) subplot was enlarged and bolded, in the hope that will help with readability. Additionally, the pymol file (rigid_core.pse, available at https://osf.io/dvzq7/) showing all the considered interactions presented in the Figure 5B has been uploaded to the SI, considering reporting around 1200 pair distance would be difficult to relate with the protein structure. Different color (color scheme in Figure 3B) represents the standard deviation of the distance variance during the simulation.

17. - I'm not sure that the data reported in figure 5F are statistically significant. What about these data in the single variants? Are these data similar in the six replicas for each system?

As we noted above, we have added text to highlight our reasoning for analyzing and presenting aggregated data only. We plot these data in the single variant, the similar trend is still show in D170A variant. While in the six replicas, the data is not similar as we observed in this pair RMSD, some trajectories undergo transmission between different minimums, some of which is in a stable minimum. For transparency, we have included this figure as Figure S7.

18. - Are REDAN analyses performed on both the proteins (wild type and D170A variant)?

Yes, the relative entropies of the pair distance distributions between wild type and D170A variants are used as the weight of the edge of the graph.

19. - the color code used in figure S4F is not clear

We apologize for lack of the note of color code, it has been added in the figure S4F(updated to Figure S8F).

20. - in the discussion, the authors write that " The D170A variant explores and extends conformational space of SH2 domain, specifically with significant changes in opening of the pY+3 pocket.". SASA data could be useful to strengthen this statement

We totally agree with reviewer’s comment, unfortunately, we didn’t see any reported data regarding it, and we don’t have the facility to do it.

21. - in the methods section or in the figure 1 caption an explanation of the method used to obtain the structure in figure 1 should be inserted

Thanks for the suggestion, the information has been added in the method section.

“To show DNA binding (Figure 1), the 1BG1 structure was used which contains a single STAT3 bound to DNA. However, STAT3 binds to DNA in its dimeric state; the dimeric structure was generated in PyMol to show DNA binding of STAT3 dimer.”

**Reviewer #3**

In this manuscript, authors investigated the structural and dynamic features of wild type and mutated STAT3 protein using molecular dynamics simulations. The comparison between the wild and mutated simulations provided not only the structural and dynamical difference but also the information on allosteric mechanism. This work is interesting to the readers of PLOS computational biology, it would strengthen the paper if the authors could address the following points.

Major points:

1. In this paper, the result and discussion parts are separated. The results include inferences based on the other research; I think it is okay to include such descriptions. However, some of the descriptions seems to hinder readability. For example, P13 line 4-5 “the rigidity transmission was observed in other proteins (Sljoka 23 , Ye et al. 24 ). Thus we further hypothesized…”. I found some of the explanations in the result part difficult to follow; as I suspect a reader less familiar with this protein might have even greater difficulties, authors should make the manuscript and the figures easier to understand for readers.

Thanks for the suggestion, we have elaborated this sentence to better reflect the meaning.

“The rigidity transmission, where a perturbation of rigidity at one binding site can be allosterically transmitted to a second distant site, was observed in other proteins (Sljoka (2020); Ye et al. (2018)). Thus, we further hypothesized”

Besides, we also make several other changes with the text and figures regarding other reviewers’ comments, hope that will enhance the readability.

2. The authors should correct the title of result section more appropriately.

Thanks for the suggestion, we did minor changes to enhance the readability.

3. P6-, the authors also analyzed the whole structural change. I suggest that a domain analysis (for example, dyndom, etc) using those representative structures may support your results.

Thanks for the suggestion. It is a great tool to know and adopt in future other studies. However, we think it is not appropriate in our case. dyndom require the interdomain deformation is at least comparable to the intradomain deformation. However, in our case, the whole protein is like a rigid backbone, and interdomain changes are subtle.

4. P9, line 4-7, the sentence “From these qualitative observations, it can be inferred that the increased flexibility in CCD due to loss of a negatively charged residue D170, leads to an allosteric increase in flexibility of the DBD and a decrease in flexibility of the LD and SH2 domains.” is difficult to understand. They may add a more detailed explanation based on the results of RMSD and RMSF.

Thanks a lot for pointing it out. After revisiting, we apologize for the pre-mature sentence, we have deleted from the main text.

5. P11 line 2 and Figure S2C, I understand that “The ten largest coefficients of the first two PCs” means the eigenvectors of PCA. Is this okay? If so, is it correct that these values of Figure S2C positive values? (Absolute values?)

Yes, the coefficients are the eigenvectors of PCA, the absolute value of PC1 and PC2, and square root of sum of squared PC1, PC2 values are shown here. A note has been added to the caption of Figure S2C (updated to Figure S5C)

6. P11 line 18, in this sentence “Besides, Zhang et. al. observed that the truncation of STAT3 to exclude α1 helix is fully capable of DNA binding upon tyrosine phosphorylation by Src kinase in vitro, suggesting the preservation of a functional conformation of the pY pocket. This agrees with our observation of a stable pY pocket in the D170A variant”, can the author explain the relationship between pY+3 pocket structure in simulations and this experimental data?

We are grateful for the reviewer to point this out, after revisiting, we realized this deduction does not stand. In the experiment, they delete the α1 helix. We did the simulation on D170A variant. Two system are very different. Thus, we decide to delete the sentence.

7. P12 last part -P13, I think that the sentence “Significant differences in global helical tilt for each of the macro-states of the SH2 domain is observed in α3 showing functional correlative differences among macro-states (Figure 5H). The α3 helix also interfaces with most of the other domains through its C-terminal helical turn. Residues at this interface could transmit motion through interacting residues from other domains.” is very important point. Does this data (Figure 5H->F?) show statistically reliable differences? Authors should add a more detailed explanation on this.

To quantify the statistical differences, we conducted the Kolmogorov-Smirnov test and T-test, results are shown in the table below (also included in supplement as Table S2). According to these results, we can conclude that the differences among the distributions shown in Figure 5F are statistically significant. And this Table has been included in the supplement (Table S2). Also noted in the caption of Figure 5(updated to Figure 3).

“Statistical tests (T-test and Kolmogorov-Smirnov test) are shown in Table S2.”

|  | Kolmogorov-Smirnov test | | T-test | |
| --- | --- | --- | --- | --- |
|  | statistic | P-value | statistic | P-value |
| M0 vs M1 | 0.198308 | 2.13E-75 | 20.19042 | 3.63E-88 |
| M0 vs M2 | 0.475441 | 0.336959 | 57.96138 | 0 |
| M0 vs M3 | 0.265266 | 2.78E-15 | 26.0582 | 1.41E-142 |
| M0 vs M4 | 0.366955 | 0 | 49.1721 | 0 |
| M1 vs M2 | 0.33437 | 0.005855 | 44.35194 | 0 |
| M1 vs M3 | 0.074214 | 8.72E-14 | 6.581899 | 4.85E-11 |
| M1 vs M4 | 0.189818 | 4.47E-168 | 32.10457 | 8.15E-213 |
| M2 vs M3 | 0.298892 | 1.37E-13 | -38.6448 | 2.03E-306 |
| M2 vs M4 | 0.161786 | 1.47E-117 | -26.4637 | 8.43E-148 |
| M3 vs M4 | 0.143099 | 2.57E-81 | 24.44831 | 1.35E-126 |

8. P13 line 7-9, in the sentence “The existence of such an interaction network is demonstrated in Figure 5B, where the inter-domain pair Cα distances are plotted and colored according to standard deviation values computed across all trajectories”, I suggest that authors may add a more detailed description on analysis of inter-domain pair Ca distances.

We thank the reviewer for bringing our attention on the lack of detailed description on analysis of inter-domain pair Ca distances. A detailed description of inter-domain pair Ca distances has been added in the method section.

“Inter-domain pair Cα distances

The inter-domain pair Cα distances were used to carry out the rigid core analysis. Take CCD-DBD domain pair Cα distances for example, first, the neighbors Cα atoms from DBD that are within 1 nm of CCD were found using compute_neighbors function from MDTraj, from which we can find the pair residues between CCD and DBD that are within interaction ranges. then the common pair residues between wild type and D170A variant were kept, and the pair ca distance were calculated using compute_distances from MDTraj.”

9. In methods (P23~), the authors should add a more detailed description (number of water molecules, etc) and figure on the simulation system. In addition, they should also add the figure to explain locations of the D170A, pY pocket, and pY+3 pocket

Thanks for the suggestions, a more detailed description has been provided, along with a figure depicting the simulation system. The D170A position has been added in Figure 1A, and the PY pocket and PY+3 pocket are shown in Figure 4D.

10. P23, why did the authors perform NVT simulations as production runs.

Usually, there is quite small difference between NVT and NPT. However, NVT ensemble provides the advantage of less perturbation of the trajectory, due to the absence of coupling to a pressure bath. A note has been added to the Methods section.

11. P24, the authors should add a more detailed description of cross-correlation and RMSF (how to fit, etc). The information is very important to understand the results.

Thanks for your suggestions. The cross-correlation was done by merging the rmsd of 6 copies trajectory together, and then the Pearson correlation among different domains were calculated. This information has been added to the caption of Figure S3. And A more detailed description of RMSF has been added to the method section of RMSF.

“The trajectories were first superposed to the backbone of first frame of each trajectory, then the RMSF values for each trajectory were measured using mdtraj.rmsf using MDTraj. Finally, the mean RMSF values and the standard error of each residue over the six replicas were calculated and plotted.”

**Minor points**

I think that there are several mistakes in writing in this manuscript (see below). Please check out the manuscript.

1. P23, Molecular dynamics simulation, “3ms”  “3 μs” ? It causes misunderstanding.
2. P3, amino-terminal domain()
3. P23, initial structure, “6TLE””6TLC”?
4. authors should define abbreviation of interleukin (IL)
5. P12, Figure 5H 5F.
6. P14, authors should add the descriptions on M0-M6 in caption of Figure 5.
7. P14, Figure 5B, the figure and letters are too small to understand.

Thanks for the kindly pointing out, above errors have been fixed.
