# Supplementary material for: Allosteric regulation in STAT3 interdomains is mediated by a rigid core: SH2 domain regulation by CCD in D170A variant
Source: PLoS Comput Biol. 2022 Dec 21;18(12):e1010794. doi: 10.1371/journal.pcbi.1010794 (PMC9815575; doi:10.1371/journal.pcbi.1010794)
Supplement: Attachment — Submitted filename: response.docx [file pcbi.1010794.s017.docx]

Dear Prof. Gallo,

I would like to again thank the reviewers for their thorough and constructive comments. Because reviewers 1 and 3 are satisfied with the current manuscript, we focus our attention on reviewer 2. We have worked to address this reviewer’s “central questions” as well as the more minor changes requested in the revised manuscript and attached response. The reviewer focuses on two such questions: whether the sampling undertaken is sufficient and truly representative of the overall dynamics, and whether the proposed allosteric mechanism is supported by the data (specifically, whether the conformational changes and tilt of the CCD helices are sufficiently significant). We believe an affirmative answer to both questions is supported by the analyses in the paper and by the specific responses given below. A separate version of the manuscript with changes highlighted has been provided for convenience. We hope that the manuscript is now ready for publication in PLOS Computational Biology.

Sincerely,

Devin Matthews

[damatthews@smu.edu](mailto:damatthews@smu.edu)

Reviewer #2: The general quality of the revised version of the paper “Allosteric Regulation in STAT3 Interdomains is Mediated by a Rigid Core: SH2 Domain Regulation by CCD in D170A Variant” from Devin Matthews and co-workers is surely improved with respect to the previous version, and many concerns raised from the reviewers, mine included, have been faced and solved. However, the new data leaves unanswered two central questions.

1. First, are the six simulations truly representative of the protein dynamics?

We have found a potential allosteric pathway for modification of SH2 binding affinity due to CCD modification which is plausibly supported by the data. Are alternative mechanisms possible given additional sampling? Possibly, although we do not think it likely, and the existence of alternative mechanisms does not discount the plausibility of the mechanism presented.

There are two main reasons that we did not pursue additional sampling: first, we believe that the data so far obtained plausibly support the proposed mechanism as a possible route for SH2 binding affinity modification starting from CCD. Is it possible that other mechanisms exist, and that the “true” mechanism for experimentally observed inhibition may differ? Yes: we do not discount this possibility in the manuscript. However, the mechanism inferred from our analysis is consistent with and supported by known biological data. Notably, even if additional allosteric pathways may exist, the fact that the pathway we have identified aligns with known mechanistic roles of the protein relevant to drug discovery is of merit, and identifies a plausible way to target STAT3 with small molecules. Second, we did not feel that the expense of additional simulations was justified given the obtained results and given limits on computational resources.

We attempted to be very cautious with our data analysis and the conclusions drawn from what we observed. In order to further clarify the limits of our data and conclusions, we added the following text to the manuscript:

“Since the present MD simulation cannot guarantee sampling of the full conformational space of the protein, other possible allosteric pathways cannot be excluded. The mechanism proposed here is drawn with caution and the results agree with experimental observations. Thus, this work presents a theoretical foundation upon which to draw inspiration for drug design as well as mechanistic studies on the protein.”

2. And second, is the mechanism connecting the tilt of the α3 helix in the CCD and the dynamics of the pY+3 pocket in the SH2 domain rigorously demonstrated? Let me start with the second question.

- I think that figure S7, mainly the B panel (that above on the right, as letters are reported in the caption but not in the figure) clearly shows that this correlation does not exist. In all the states the tilt of the helix populates the same distribution (with a slight exception for the distribution in the M0 state)

Sub-figure letters have been added to S7 (now S4). We agree with that the correlation seems to be extremely subtle in the aggregate data presented. However, these small differences are correlated to larger motions when we study the structural dynamics. The difference is small but causes a cascading effect at the effector region. Take the following structures as example, small rotation of Ile252 (located at α3) could shift Gln511 (located at α21) because of the conserved the hydrogen network, which end up pushing away ILE569(located at α26) from β22. The detailed hydrogen bond analysis as well as REDAN analysis was performed precisely to address a lack of distinct correlation. These analyses help us tease out the subtle allosteric events that lead to large conformational changes in the SH2 domain.

- and I really don’t understand how the P-values reported in table S2 can reach so low numbers (e.g. 2.03E-306, between M2 and M3), how many freedom degrees have been considered? By the way, but this is a minor point, I definitively prefer to distinguish between p < 0.05 for statistically significant correlations and < 0.001 for highly significant ones, (What is the real difference between 2.03E-306 for M2/M3 and 0 for M1/M2?).

We have added the degree of freedom information for the T-test in Table S2 and cleaned up the small P-values. Indeed the P-values are quite small, but result from standard statistical tests and, in our opinion, are reliable given the large sample size.

|  | Kolmogorov-Smirnov test | | T-test | | |
| --- | --- | --- | --- | --- | --- |
|  | statistic | P-value | Degree of freedom | statistic | P-value |
| M0 vs M1 | 0.198308 | <0.001 | 7028 | 20.19042 | <0.001 |
| M0 vs M2 | 0.475441 | 0.336959 | 6882 | 57.96138 | <0.001 |
| M0 vs M3 | 0.265266 | <0.001 | 6610 | 26.0582 | <0.001 |
| M0 vs M4 | 0.366955 | <0.001 | 3931 | 49.1721 | <0.001 |
| M1 vs M2 | 0.33437 | 0.005855 | 12019 | 44.35194 | <0.001 |
| M1 vs M3 | 0.074214 | <0.001 | 11219 | 6.581899 | <0.001 |
| M1 vs M4 | 0.189818 | <0.001 | 7990 | 32.10457 | <0.001 |
| M2 vs M3 | 0.298892 | <0.001 | 10969 | -38.6448 | <0.001 |
| M2 vs M4 | 0.161786 | <0.001 | 7725 | -26.4637 | <0.001 |
| M3 vs M4 | 0.143099 | <0.001 | 6799 | 24.44831 | <0.001 |

The P-values are calculated based on Figure 3F. The mean and the standard deviation and the shape of the distribution are quite different among different macrostates. Indeed, the standard deviation alone does not clearly separate the distributions. However, the large number of samples in each population lead to a vanishingly small (in most cases) probability that the populations are in fact drawn from the same distribution.

| Macrostate | Mean | Standard deviation |
| --- | --- | --- |
| 0 | 28.37534 | 2.895679912 |
| 1 | 27.11379 | 2.944798157 |
| 2 | 24.77636 | 2.83295476 |
| 3 | 26.77098 | 2.576996914 |
| 4 | 25.82367 | 2.823122524 |

- In addition, also by looking at figure 4, it seems that the conformation of the α3 helix is the same in the five states. I think that the authors need to search for more robust pieces of evidence of the correlation between α3 helix and SH2 dynamics, otherwise, different explanations have to be proposed. The sentence “The rigidity of the α helices diminished the utility of PCA analysis”, is surely true for inter-domain PCA, but I think that PCA calculated on a sub-space (e.g the α3 helix in the CCD domain and the SH2 domain could enforce the proposed picture) could be helpful.

In figure 4, the structures were aligned to each other with respect to the Cα, thus largest portions of structure appear to be overlayed onto each other. However, we see a distinct differences in the tail of multiple alpha helices, indicating a difference in angular tilt of the helices (in addition to a “kink” in certain configurations).

We have used multiple features (dihedral angle, pair Cα distance, Cα coordinates) to describe CCD domain to perform PCA analysis, the results consistently show low variance. For clarity, the quoted sentence “The rigidity of the α helices diminished the utility of PCA analysis” has been updated to:

“The rigidity of the α helices diminishes the utility of PCA withing the CCD alone to study intradomain conformational differences.”

We feel that this is indeed evidence that any changes in the CCD must therefore occur through rigid body allostery. The changes in α3 helix are small, the variances would mainly from SH2 domain, thus the PCA results of both SH2 domain and CCD domain would be no different from PCA of SH2 domain alone.

- Beyond this aspect, in my opinion, concerns remain on the efficacy of sampling. The authors write (page 9 line 276): “The long transition timescale between these states validates our kinetic clustering model, but also necessitates a “global” view of the trajectories in order to explain the full conformational dynamics of the protein". If long transition timescales characterize the system, the importance of each state is truely difficult to be predicted, as also cannot be ruled out that other states (not sampled in the six simulations) play a role. Furthermore, if stochastic transitions characterize the dynamics in the simulations, how can the authors exclude the possibility that wild-type will populate the M0, M1, and M3 states in further simulations? Probably for so complex system, enhanced sampling techniques could be preferable.

Please see the response to question 1. Enhanced sampling techniques is an interesting point that we have considered during our course of the study. However, enhanced sampling techniques must be paired with rigorous experimental design to eliminate bias due to external artificial potential applied. Thus, we determined that the information obtained from the presented simulation is a valuable perspective towards understanding STAT3 allostery. We believe further experiments like peptide specificity binding assay by mutating residues that build the rigid core would be better suited to verify the mechanism we proposed here as well as identify possible alternative mechanisms.

A few minor points remain:

1) In the answer to my question 20, in which I asked for SASA analysis, the authors answer that “we don’t have the facility to do it”, but SASA can be calculated with different free software without particular difficulties. I invite again the authors to calculate these data.

We misunderstood the SASA acronym previously. We have now calculated the solvent accessible surface area, shown below. This additional analysis does not alter any conclusions we have discussed in the paper.

2) In the caption of figure 1 “there us” instead of “there is”

This typo has been corrected.

3) The figures in SI are recalled quite randomly.

Thank for kindly pointing this out, the supplement figures have been reindexed as its appearance order in the manuscript.

4) Page 6 line 154 “The hypothesis was tested by comparing corresponding helical tilt at the CCD of different macro-states identified in Conformational differences in the pY+3 binding pocket correlate to the decreased binding affinity from wild type to D170A variant for the pY and pY+3 pockets.” The sentence is not clear to me.

This a latex error where the full section name was referenced incorrectly. It is supposed to be:

“The hypothesis was tested by comparing corresponding helical tilt at the CCD of different macro-states identified in section ‘Conformational differences in the pY+3 binding pocket correlate to the decreased binding affinity from wild type to D170A variant for the pY and pY+3 pockets’”.

This sentence has been updated in the manuscript.

5) Caption figure 4 indicates α21, whilst in the figure α27 is highlighted

The caption has been corrected.
