# Supplementary material for: Allosteric regulation in STAT3 interdomains is mediated by a rigid core: SH2 domain regulation by CCD in D170A variant
Source: PLoS Comput Biol. 2022 Dec 21;18(12):e1010794. doi: 10.1371/journal.pcbi.1010794 (PMC9815575; doi:10.1371/journal.pcbi.1010794)
Supplement: Attachment — Submitted filename: response.docx [file pcbi.1010794.s018.docx]

Dear Prof. Gallo,

I would like to again thank the reviewers for their thorough and constructive comments. We critically examined reviewer2’s question about the significance of helix 3 tilt, and the specific responses are given below. A separate version of the manuscript with changes highlighted has been provided for convenience. We hope that the manuscript is now ready for publication in PLOS Computational Biology.

Sincerely,

Devin Matthews

[damatthews@smu.edu](mailto:damatthews@smu.edu)

Reviewer #2: In the previous revision, I had two main concerns. Concerning the first one, the authors have added a sentence of caution regarding the efficacy of the sampling; considering the complexity of the system, this can be considered sufficient.

On the contrary, practically nothing has been done to demonstrate a correlation between the movement of α3 helix in the CCD domain and the binding site on the SH2 domain.

In my opinion, data reported in figure 3F and in figure S4 show that a correlation is absent. The authors wrote that “as example, small rotation of Ile252 (located at α3) could shift Gln511 (located at α21) because of the conserved the hydrogen network, which end up pushing away ILE569(located at α26) from β22.”. This suggests that probably a correlation exists, but it does not necessarily involve the movements of the α3 helix (that is a global variable, probably less affected by small variations).

To support their statement the authors, refer to data in table S2. In this table now the degrees of freedom are reported. These numbers could be an overestimation of the real degrees of freedom; for example, it is unclear to me if the considered conformations have been sampled with an interval of time greater than the time decay of the autocorrelation of the helical tilt. If this is not the case, the considered conformations would not be completely independent (as a part of a trajectory), the real degrees of freedom could be significantly lower and, in turn, the p-values sensitively higher than those reported. In summarizing, I remain of the idea that a correlation between the α-helical tilt and the dynamics of the binding site is not demonstrated. As stated above, a correlation between some structural features in the CDD domain and the dynamics of the binding site in the SH2 domain could exist, but this correlation probably involves features more punctual than the helical tilt.

We thank the reviewer for their comments and apologize if our prior responses did not adequately address their concerns. First, we would like to note, that the helical tilt of the α3 helix is not a central part of the manuscript, nor the central feature of the proposed allosteric mechanism. While the α3 helix is an important piece of the allosteric mechanism (vide infra), we only suggest that the global tilt angle serves as an indicator of the more detailed conformational state rather than a single controlling parameter. Rather, our proposed allosteric mechanism centers on the role of a rigid core that links the terminal ends of the CCD to the SH2 domain. The rigid core propagates the initial conformational changes through α3, which is ideally situated at the junction of a lever arm between the CCD and the SH2 domain. As we state in the manuscript, it is likely that these initial conformational changes are specific to the effector used, but our findings suggest the possibility of a shared mechanism via the rigid core. We have provided rigorous analysis using PCA, rigid core analysis, and REDAN. The primary findings within 2D plots of our PCA analysis of the rigid core demonstrate two distinct conformations (Fig S4 A, B). Subsequent analysis indicated that the population of the two conformations were dependent upon interactions between residue 562 of the rigid core and the α3 helix (Fig. S4C). When these pairwise Cα distances were removed, the conformational landscape for WT and D170A are indistinguishable (Fig. S5A,B). Thus, alteration of the conformational landscape in the D170A variant hinges upon coupling subtle changes in the CCD to α3, through α21 (in LD) and α20 (in DBD) to α26 and α24 (in LD), and finally leading to allosteric modification of SH2 via β29. We believe that these primary findings are rigorously studied, are the center point of the manuscript, and are independent from a possible causal role of the α3 global helical tilt.

Our discussion of the α3 helical tilt is secondary to the above findings and is also supported by existing experimental data. Prior crystallographic studies of a monobody (MS3-6) that disrupts STAT3 function, identified that MS3-6 binds to the CCD. Further, its proposed mechanism of action was due to subtle alterations in the relative orientation of helices in the CCD. Given that the crystal structures contained packing interactions within the CCD that may alter the helical structure, we were curious as to whether we could independently verify, in the absence of complications due to crystallographic packing interactions, subtle variations in helical tilts that may impact the SH2 domain. References to this prior work have now been added and made clearer in the relationship to our study. We contend that our analysis independently confirms aspects of the previously published experimental data. We summarize our argument again here:

Markov State Modelling and Perron Cluster Cluster analysis demonstrate that the WT occupies only 2 macro states, whereas the D170A variant samples 5 macro states. Averaging across all states, the WT protein demonstrates an average helical tilt of 25.2, whereas the D170A is rotated by 26.9 degrees on average. This is to a large part due to states M0, M1, and M3 that are unique to the D170A variant having larger helical tilts than either state in the WT. Notably, even within the M2 and M4 populations that are shared by WT and D170A, the average helical tilt is shifted in the D170A population. Although a subtle rotation, these are consistent with the helical movements found in the CCD due to MS3-6 binding that impact biological function, and thus in our opinion merit discussion in the manuscript.

Regarding the reduction in degrees of freedom and statistical significance of the tilt angle distributions: the partial autocorrelatin was calculated for each trajectory, and the partial autocorrelation plot suggests that there would be no correlation for lag time beyond 1ns. The distribution as well as the Welch’s t-test were recalculated with trajectories sampled at the interval of 1ns, the degrees of freedom is significantly lower, but the p-values remain statistically significant small.

|  | Degree of Freedom | statistic | P value |
| --- | --- | --- | --- |
| M0_M1 | 661.3365 | 4.725546 | <0.001 |
| M0_M2 | 648.729 | 16.4242 | <0.001 |
| M0_M3 | 610.8947 | 7.593057 | <0.001 |
| M0_M4 | 373.8996 | 14.04471 | <0.001 |
| M1_M2 | 1198.157 | 14.03343 | <0.001 |
| M1_M3 | 1141.496 | 3.343177 | <0.001 |
| M1_M4 | 809.9727 | 11.17431 | <0.001 |
| M2_M3 | 1096.967 | 11.14801 | <0.001 |
| M2_M4 | 755.6419 | 7.358269 | <0.001 |
| M3_M4 | 702.0243 | 7.317803 | <0.001 |

As noted above, because of consistency with previously published crystal structures and biological data, we feel it is important to mention the α3 helical tilt. However, we recognize the reviewers concerns and have made several changes to the text to clarify why we conducted the helical tilt study (e.g. prior published work), and to reduce the emphasis on the helical tilt as it relates to signaling, and instead focus on the primary point of the CCD transmitting allosteric signals through a rigid core that can propagate motion through structurally-conserved residue interactions (e.g. residue 562) between α3 through α21 (in LD) and α20 (in DBD) to α26 and α24 (in LD), and finally leading to allosteric modification of SH2 via β29. The full set of changes are made to the manuscript.

Figure S3: The PCA analysis of pair distances of rigid core (residues:240-252,511,474,479,546,550,549,562,568,564,610,611). **(A)** PCA 2D plot colored by different macrostates; **(B)** PCA 2D plot colored by systems; **(C)** The first ten features that contribute the first two PC the most. The absolute value of PC1 and PC2, and square root of sum of squared PC1 and PC2 values are shown here.

Figure S4: The PCA analysis of pair distances of rigid core with residue 562 (residues:240-252,511,474,479,546,550,549,568,564,610,611). **(A)** PCA 2D plot colored by different macrostates; **(B)** PCA 2D plot colored by systems; **(C)** The first ten features that contribute the first two PC the most. The absolute value of PC1 and PC2, and square root of sum of squared PC1 and PC2 values are shown here.
